# A temporal shift of the evolutionary principle shaping intratumor heterogeneity in colorectal cancer

**DOI:** 10.1038/s41467-018-05226-0

**Published:** 2018-07-23

**Authors:** Tomoko Saito, Atsushi Niida, Ryutaro Uchi, Hidenari Hirata, Hisateru Komatsu, Shotaro Sakimura, Shuto Hayashi, Sho Nambara, Yosuke Kuroda, Shuhei Ito, Hidetoshi Eguchi, Takaaki Masuda, Keishi Sugimachi, Taro Tobo, Haruto Nishida, Tsutomu Daa, Kenichi Chiba, Yuichi Shiraishi, Tetsuichi Yoshizato, Masaaki Kodama, Tadayoshi Okimoto, Kazuhiro Mizukami, Ryo Ogawa, Kazuhisa Okamoto, Mitsutaka Shuto, Kensuke Fukuda, Yusuke Matsui, Teppei Shimamura, Takanori Hasegawa, Yuichiro Doki, Satoshi Nagayama, Kazutaka Yamada, Mamoru Kato, Tatsuhiro Shibata, Masaki Mori, Hiroyuki Aburatani, Kazunari Murakami, Yutaka Suzuki, Seishi Ogawa, Satoru Miyano, Koshi Mimori

**Affiliations:** 1 Department of Surgery, Kyushu University Beppu Hospital, 4546 Tsurumihara, Beppu, 874-0838 Japan; 2 Department of Gastroenterology, Oita University Hospital, 1-1 Idaigaoka, Yufu, 879-5593 Japan; 3 Division of Health Medical Computational Science, Health Intelligence Center, Institute of Medical Science, The University of Tokyo, 4-6-1 Shirokanedai, Minato-ku, Tokyo 108-8639 Japan; 4 Laboratory of DNA Information Analysis, Human Genome Center, Institute of Medical Science, The University of Tokyo, 4-6-1 Shirokanedai, Minato-ku, Tokyo 108-8639 Japan; 5 Department of Pathology, Kyushu University Beppu Hospital, 4546 Tsurumihara, Beppu, 874-0838 Japan; 6 Department of Diagnostic Pathology, Oita University Hospital, 1-1 Idaigaoka, Yufu, 879-5593 Japan; 7 Department of Pathology and Tumor Biology, Graduate School of Medicine, Kyoto University, Yoshida-Konoe-cho, Kyoto-shi Sakyo-ku, Kyoto, 606-8501 Japan; 8 Division of Systems Biology, Nagoya University Graduate School of Medicine, 65 Tsurumai-cho, Showa-ku, Nagoya, 466-8550 Japan; 9 Division of Health Medical Data Science, Health Intelligence Center, Institute of Medical Science, The University of Tokyo, 4-6-1 Shirokanedai, Minato-ku, Tokyo 108-8639 Japan; 10 Department of Gastroenterological Surgery, Graduate School of Medicine, Osaka University, 2-2 Yamadaoka, Suita, 565-0871 Japan; 11 Gastroenterological Center, Department of Gastroenterological Surgery, Cancer Institute Hospital, Japanese Foundation for Cancer Research, 3-8-31 Ariake, Koto, Tokyo 135-8550 Japan; 12 Department of Surgery, Takano Hospital, 4-2-88 Obiyama, Chuo-ku, Kumamoto 862-0924 Japan; 13 Department of Bioinformatics, National Cancer Center Research Institute, 5-1-1 Tsukiji, Chuo-ku, Tokyo 104-0045 Japan; 14 Division of Cancer Genomics, National Cancer Center Research Institute, 5-1-1 Tsukiji, Chuo-ku, Tokyo 104-0045 Japan; 15 Laboratory of Molecular Medicine, Human Genome Center, Institute of Medical Science, The University of Tokyo, 4-6-1 Shirokanedai, Minato-ku, Tokyo 108-8639 Japan; 16 Genome Science Division, Research Center for Advanced Science and Technology (RCAST), The University of Tokyo, 4-6-1 Komaba, Meguro-ku, Tokyo 153-8904 Japan; 17 Laboratory of Systems Genomics, Department of Computational Biology and Medical Sciences, Graduate School of Frontier Sciences, The University of Tokyo, 5-1-5 Kashiwanoha, Kashiwa-shi, Chiba 277-8561 Japan

## Abstract

Advanced colorectal cancer harbors extensive intratumor heterogeneity shaped by neutral evolution; however, intratumor heterogeneity in colorectal precancerous lesions has been poorly studied. We perform multiregion whole-exome sequencing on ten early colorectal tumors, which contained adenoma and carcinoma in situ. By comparing with sequencing data from advanced colorectal tumors, we show that the early tumors accumulate a higher proportion of subclonal driver mutations than the advanced tumors, which is highlighted by subclonal mutations in *KRAS* and *APC*. We also demonstrate that variant allele frequencies of subclonal mutations tend to be higher in early tumors, suggesting that the subclonal mutations are subject to selective sweep in early tumorigenesis while neutral evolution is dominant in advanced ones. This study establishes that the evolutionary principle underlying intratumor heterogeneity shifts from Darwinian to neutral evolution during colorectal tumor progression.

## Abstract (web summary)

Advanced colorectal cancers are characterised by intra-tumour heterogeneity dictated by neutral evolution. Here the authors analyse early colorectal tumours by whole-exome sequencing and find that Darwinian evolution determines the fate of early lesions in colorectal adenoma and carcinoma in situ.

## Introduction

Cancer evolution and intratumor heterogeneity (ITH) have attracted increasing attention in the cancer research field because ITH generated during cancer evolution presumably contributes to the therapeutic and diagnostic difficulties of cancer. With the advent of next-generation sequencing technology, the multiregion sequencing approach has been popularly used to understand ITH. Multiregion sequencing, in which multiple samples from physically separate regions of a single tumor are sequenced, typically identifies two categories of somatic mutations: “ubiquitous” and “heterogeneous” mutations, which are present in either all regions or a subset of regions, respectively. Ubiquitous mutations are assumed to accumulate in the early phase of cancer evolution. The parental clone that has acquired all the ubiquitous mutations then branches into subclones, which accumulate heterogeneous mutations and shape ITH. Multiregion sequencing has revealed the landscapes of ITH for renal^1,2^, breast^3^, esophageal^4,5^, lung^6,7^, ovarian^8^, prostate^9,10^, pancreatic^11^, and other types of cancer. These studies have presented evidence that Darwinian evolution shapes at least part of ITH: there exist one or more subclonal driver events within distinct subclones of a tumor (hereafter, this evidence will be referred to simply as branched evolution). For a few types of tumors^1–3,5^, more convincing evidence has been identified: multiple subclones harbor genetic alterations in the same gene or genes that work in the same pathway (hereafter, referred to as parallel evolution).

In the development of colorectal cancer (CRC), adenoma first forms a polyp and then partially progresses to early carcinoma, which subsequently grows beyond the muscularis mucosa to invade surrounding tissues and finally metastasize^12^. To examine ITH in advanced CRC (ACRC), we previously performed multiregion sequencing of nine locally advanced or metastatic tumors^13^. While most of the known driver events represented by *APC* and *KRAS* mutations were observed as ubiquitous mutations, branched or parallel evolution was rarely observed in evolutionary histories of ACRC. By additionally performing a computational simulation of cancer evolution, we demonstrated the possibility that ITH in ACRC could be generated by neutral evolution. Other studies similarly combined multiregion analysis and mathematical modeling to report that neutral evolution could shape the majority of ITH in CRC as well as liver cancer^14,15^. The neutral evolution model was also reported by analyzing the distribution of variant allele frequencies (VAFs) in single-region sequencing data^16,17^.

Although differences in ITH across various cancer types have been well studied, little has been reported on the changes in ITH along the time course of tumorigenesis. To investigate ITH in the early process of colorectal tumorigenesis, we performed multiregion sequencing of ten colorectal tumors containing adenoma and early carcinoma. In contrast to our previous report about ACRC^13^, our multiregion analysis of the ten early colorectal tumors strongly suggests that Darwinian evolution plays a critical role in shaping ITH in the early phase of colorectal tumorigenesis.

## Results

### Multiregion sequencing of ten early colorectal tumor cases

**Fig. 1:**
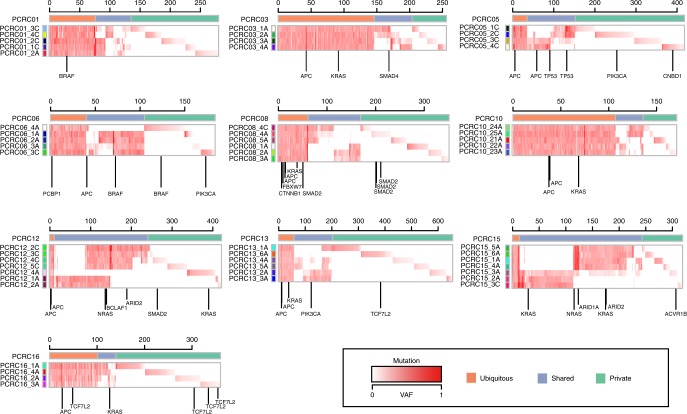
Multiregion mutation profiles of PCRCs. Ten PCRCs were subjected to multiregion WES, and VAFs of all mutations including short indels are presented as a heat map for each case. Top colored bars indicate three categories of mutations: ubiquitous, shared, and private. Left colored bars represent sample labels, which are shown such that color similarity represents similarity between mutation profiles. Previously reported driver genes with possible functional mutations, including non-synonymous SNV, stop-gain SNV, splicing SNV, or indel, are provided under each heat map. The last characters of sample names, “A” or “C”, represent the pathologic features “adenoma” or “carcinoma”, respectively

To characterize ITH in the early phase of colorectal tumorigenesis, we performed multiregion whole-exome sequencing (WES) on ten early colorectal tumor cases, the details of which are provided in Supplementary Data 1. Although the samples subjected to our analysis contained colorectal adenoma and carcinoma in situ, we collectively refer to them as precancerous lesions of colorectal cancers (PCRCs) in this study. We selected tumors that were diagnosed as colorectal laterally spreading tumors (LSTs), which have suitable forms for multiregion sampling. For each case, we sequenced four to seven multiregion tumor samples and a paired normal mucosa sample as a control, which amounted to 53 tumor samples and 10 normal samples in total. Our WES, which had a median fold coverage of 132.0 (range: 75.5–200.1), detected a median of 150 (range: 82–244) mutations for each sample (Fig. 1, Supplementary Fig. 1a, Supplementary Data 2 and 3). From this, we estimated that each sample had a median mutation rate of 3.0 (range: 1.6–4.9) mutations per megabase. Considering that eight non-hypermutated ACRCs in our previous study^13^ harbored a median of 2.8 (range: 1.2–4.8) mutations per megabase (Supplementary Figs. 1b and 2), PCRCs and ACRCs have comparable somatic mutation rates. Our hierarchical Bayesian analysis, which removed the residuals associated with samples and cases, also confirmed that there were no clear differences in the distribution of the corrected mean numbers of somatic mutations between adenoma, early carcinoma, and ACRC (see Methods; Supplementary Fig. 1c). Based on multiregion mutation profiles (Fig. 1), mutations were categorized as either ubiquitous or heterogeneous mutations. In this study, heterogeneous mutations were further subcategorized into shared mutations, which existed in some of the samples, and private mutations, which were observed in a single sample. PCR-based deep sequencing of randomly sampled mutations validated 100%, 100%, and 94.2% of ubiquitous, shared, and private mutations, respectively. We also compared the number of ubiquitous and heterogeneous mutations between PCRC and ACRC after correcting for different number of samples across cases by downsampling (see Methods). PCRC tended to harbor fewer ubiquitous mutations and more heterogeneous mutations than ACRC; particularly, the number of shared mutations was significantly large in PCRC (Supplementary Fig. 1d–f; *P* = 0.011; Wilcoxon rank-sum test). We did not observe any significant differences in mutation spectra between ubiquitous and heterogeneous mutations across ten PCRCs (Supplementary Fig. 3a; Wilcoxon signed-rank test) or between PCRC and ACRC (Supplementary Fig. 3b; Fisher’s exact test).

### Evolutionary histories of ten PCRCs

**Fig. 2:**
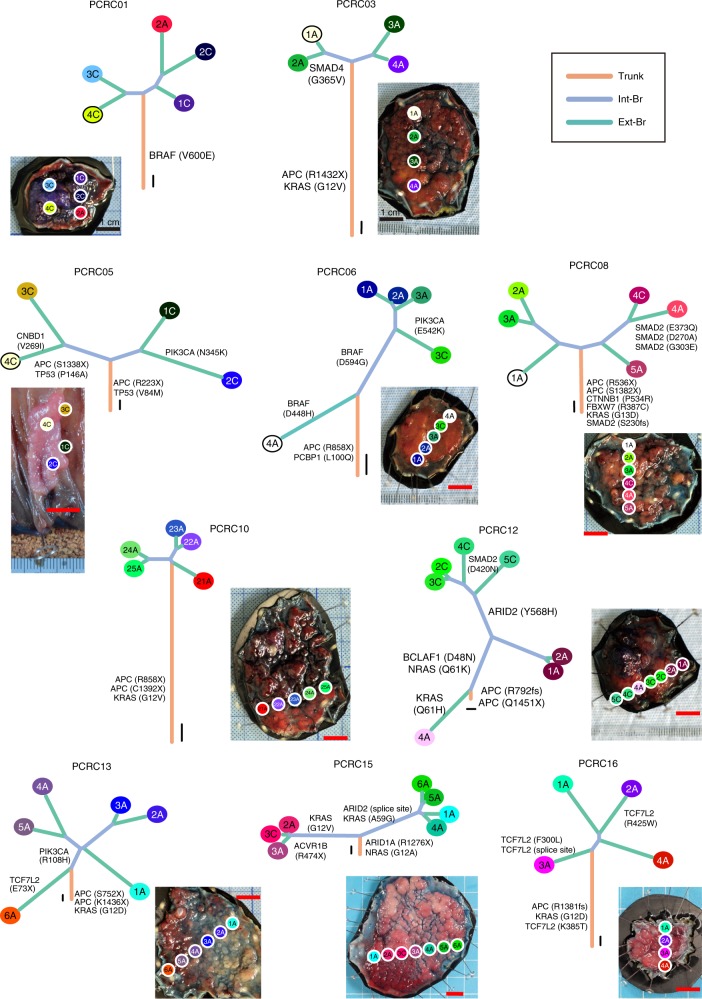
Evolutionary trees of PCRCs. Ten evolutionary trees were constructed from the multiregion WES data using the Treeomics algorithm. Trunks, internal branches (int-Br), and external branches (ext-Br) generally correspond to ubiquitous, shared, and private mutations, respectively, while leaves correspond to samples. The colors of the leaves are the same as the sample labels in Fig. 1. Lengths of the trunk and branches represent the number of mutations, and scales for ten mutations are shown near the roots of the evolutionary trees. Driver genes with possible functional mutations are mapped along the evolutionary trees. The photo of each tumor is provided with positions from which each sample was obtained. Red scale bars for one centimeter attempted with each photo

Ten PCRCs had already acquired many non-silent mutations in known CRC driver genes^18^ such as *APC*, *KRAS*, *PIK3CA*, *FBXW7*, *SMAD4*, and *TP53* (observed in 8, 7, 3, 1, 1, and 1 patients, respectively; Fig. 1). Mutation rates of *APC*, *KRAS*, *PIK3CA*, *FBXW7*, and *SMAD4* were consistent with previous reports on typical CRC^18,19^ (Supplementary Tables 1 and 2), while the mutation rate of *TP53* in PCRCs was less than that in the TCGA cohort^18,19^ (Supplementary Table 2; 10% vs. 52.4%; *P* = 0.009; Fisher’s exact test). This was partly due to higher proportion of granular-type LST cases in our cohort, which was reported to harbor lower frequency of *TP53* mutation compared to other CRC subtypes^20^. We obtained evolutionary trees of the ten PCRCs by applying the Treeomics algorithm^21^ to our multiregion sequencing data (Fig. 2). While constructing an evolutionary tree, Treeomics corrects potential sequencing artifacts so that all mutations have mutation patterns compatible with the topologies of the evolutionary tree. Based on this property, Treeomics produces a new categorization of mutations based on parts of the inferred tree; namely, we obtained “trunk” and “branch” mutations, which were refined versions of ubiquitous and heterogeneous mutations, respectively. Similarly, shared and private mutations were mapped to “internal branch” and “external branch” mutations, respectively (hereafter, the two categorizations are referred to as the ubiquitous-heterogeneous and the trunk-branch categorizations; Supplementary Fig. 4). Treeomics also employs bootstrapping analysis, which demonstrated the robustness of our evolutionary tree inference (Supplementary Fig. 5). In each evolutionary tree, the length of the trunk and branches represented the number of trunk and branch mutations, respectively. Some of the PCRC trees had “palm tree-like” shapes that were composed of long trunks and short branches; such trees were typically observed in the ACRC trees, which were reconstructed from our previously published data using Treeomics (Supplementary Figs. 6, 7 and 8a). However, five PCRC trees had “forked tree-like” shapes, which were composed of short trunks and long branches and were not observed in ACRC cases (Supplementary Fig. 8c). These data are consistent with the observation that PCRC harbored more heterogeneous mutations than ACRC (Supplementary Fig. 1d–1f). To scrutinize the evolutionary history of each tumor, we mapped known driver genes with possible functional mutations along the evolutionary trees, which contained non-synonymous single-nucleotide variants (SNVs), stop-gain SNVs, splicing SNVs, or insertion/deletions (indels). For example, PCRC05 had two major branches, which appeared in the relatively early stage of evolution. The first *APC* mutation (R223X) was found in the trunk, while the second *APC* mutation (S1338X) was found only in the left major branch. We also found that both *APC* mutations in the left major branch had VAFs of ~0.4, while the first *APC* mutation (R223X) in the right major branch had an allele frequency of ~0.8. These observations suggest the two major subclones were subjected to two different processes leading to biallelic inactivation of *APC*; an additional mutation on the second allele was acquired in the left major branch, while LOH accompanying the first mutation occurred in the right major branch. Notably, the evolutionary tree of PCRC15 showed that two major branches accumulated multiple non-silent mutations in known driver genes; the right major branch had *KRAS* (A59G) and *ARID2* (splice site), whereas the left major branch had *KRAS* (G12V) and *ACVR1B* (R474X). PCRC12 had an extremely short trunk containing double mutations in *APC* (Q1451X and R792fs) and long branches accumulating mutations on five different genes. In this case, an *NRAS* mutation (Q61K) was obtained as an internal branch mutation after the first branching point, while a *KRAS* mutation (Q61H) was obtained as an external branch mutation at the other side of the branching point. Comparisons between the evolutionary tree and physical positions of each sample suggest that subclonal branching generally proceeded in physically correlated ways. Treeomics optionally performs detection of subclonal mixing between evolutionally separated samples; our analysis detected subclonal mixing in seven of the ten cases (Supplementary Fig. 5).

### Comparative analysis of ITH between PCRC and ACRC

**Fig. 3:**
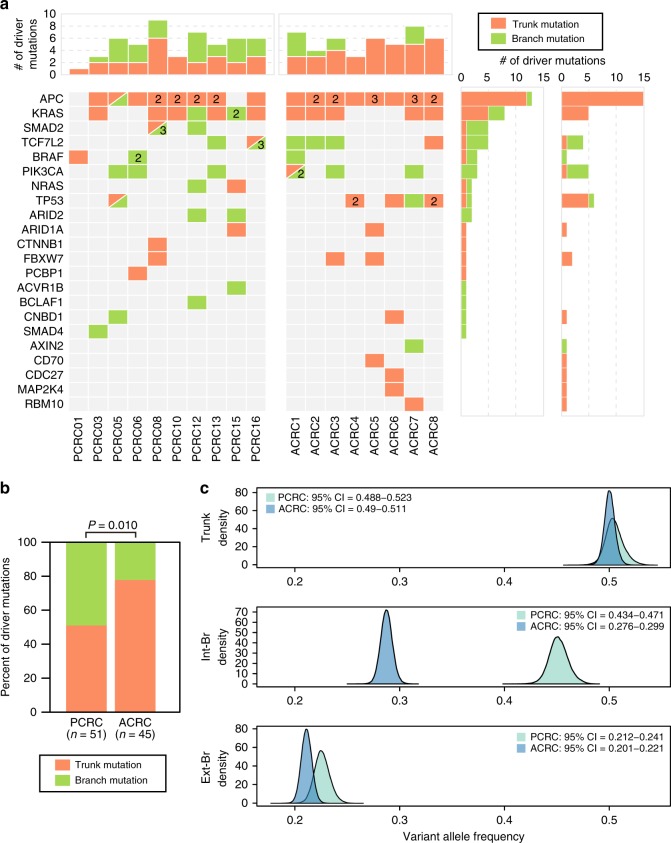
Darwinian evolution mainly shapes ITH in PCRC. The multiregion mutation profiles of the ten PCRCs were compared with those of eight non-hypermutated ACRCs; these ACRCs in our previous study led us to conclude that ITH was mainly generated by neutral evolution. **a** Distribution of driver genes. Colored tables show the presence of trunk (orange) or branch (green) mutations on known driver genes in each case of the PCRCs and ACRCs. If a case had multiple driver mutations, the number is provided within the corresponding cell. Top and right bar graphs represent the sums of driver mutations for each sample and each driver gene, respectively. **b** Bar plots showing the proportions of trunk mutations versus branch mutations on driver mutations. Significant enrichment of branch mutations on driver genes in PCRC (25/51) was compared with ACRC (10/45; *P* = 0.010; Fisher’s exact test). **c** Comparison of VAFs for trunk, internal branch (int-Br), and external branch (ext-Br) mutations. Hierarchical Bayesian analysis was employed to correct the effects of tumor content and read depth as well as to remove the residuals associated with individual mutations, samples, and cases (see Methods and Supplementary Fig. 17). The density plot shows an estimated posterior distribution of the corrected mean VAFs for trunk mutations, int-Br mutations, and ext-Br mutations in PCRC or ACRC. PCRC harbored int-Br mutations with higher VAFs than ACRC. 95% CI 95% credible interval

These evolutionary trees suggest that branched evolution and parallel evolution are prominent in PCRC evolution, which is in contrast to the result from our ACRC study^13^. To consolidate this finding, we directly compared the clonal distribution of driver mutations between PCRC and ACRC; in the 10 PCRC cases, 25 of 51 driver mutations were branch mutations, while only 10 of 45 driver mutations were branch mutations in the 8 ACRC cases (Fig. 3a). Thus, compared with ACRC, PCRC had a stronger tendency to acquire driver mutations as branch mutations (Fig. 3b; *P* = 0.010; Fisher’s exact test). When examined on the ubiquitous-heterogeneous categorization, this tendency was more statistically significant (Supplementary Fig. 9a, b; *P* = 0.00090; Fisher’s exact test), which reflects the fact that several heterogeneous driver mutations were judged as trunk driver mutations by Treeomics. The contribution of natural selection to ITH can also be measured by the distribution of VAFs; if a set of subclonal mutations consisted of driver and associated passenger mutations, natural selection should have made their allele frequencies high, compared with those from a set without driver mutations^22^. Based on this idea, we compared the distribution of VAFs between PCRC and ACRC, for each type of mutation on the trunk-branch categorization. To correct the effects of tumor content and read depth, as well as to remove the residuals associated with individual mutations, samples and cases, we employed hierarchical Bayesian analysis, which demonstrated that PCRC harbored internal branch mutations at clearly higher VAFs than ACRC (see Methods; Fig. 3c). We confirmed the same tendency in cancer cell fractions (CCFs), which were obtained by removing effects of copy number alterations (CNAs) from VAFs (see Methods; Supplementary Fig. 10a). Analysis on the ubiquitous-heterogeneous categorization also reproduced the same result, although with less clearness (Supplementary Figs. 9c and 10b). Collectively, these results strongly suggest that evolutionary principles underlying ITH substantially differ between PCRC and ACRC; Darwinian evolution plays a more critical role in generating ITH in PCRC than in ACRC.

### Multiregion analysis of CNAs

**Fig. 4:**
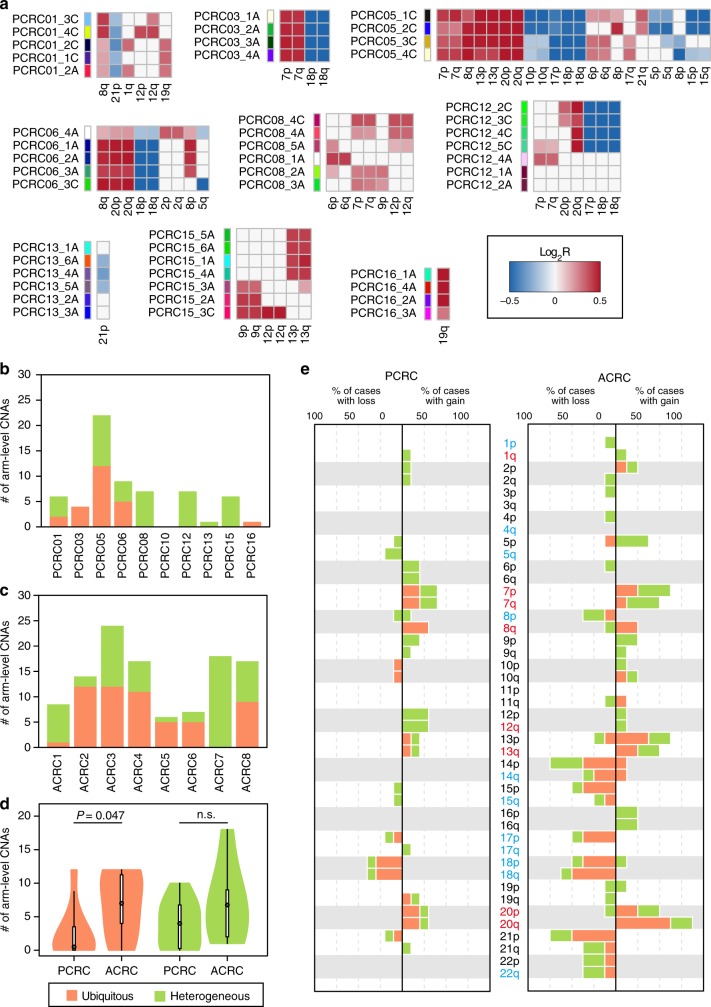
Multiregion analysis of CNAs. **a** Multiregion CNA profiles of PCRCs. Chromosomal arm-level CNAs were called from the WES data of the ten PCRCs. Heat maps represent the presence of chromosomal arm-level CNAs (red, gain; blue, loss) for each case, and the shades of color are proportional to log_2_-scaled ratios between normalized tumor and normal read depths (log_2_R). PCRC10, in which no CNAs were detected, was omitted. Samples in each case are sorted in the same order as in Fig. 1. **b, c** Bar plots showing the number of ubiquitous and heterogeneous CNAs in each case of the PCRCs (**b**) and ACRCs (**c**). Effects of different number of samples between cases were corrected by downsampling (Methods). **d** Violin plots showing the distribution of the number of ubiquitous and heterogeneous CNAs based on **b** and **c**. ACRCs harbored a significantly larger number of ubiquitous CNAs than PCRCs (*P* = 0.047; Wilcoxon rank-sum test), while the number of heterogeneous CNAs in ACRCs is comparable to that in PCRCs (*P* = 0.16; Wilcoxon rank-sum test). **e** Bar plots showing the frequencies of ubiquitous (orange) and heterogeneous (green) CNAs for PCRCs and ACRCs. For ACRCs, CNAs were called from our previously published WES data of the eight non-hypermutated ACRCs

Finally, we estimated CNAs from WES data and comparatively analyzed multiregion CNA profiles between PCRC and ACRC (Supplementary Fig. 11a–11d). In contrast to single-nucleotide mutations, our hierarchical Bayesian analysis demonstrated that the number of CNAs increased during progression from adenoma through early carcinoma to ACRC (Supplementary Fig. 11e), consistent with previous studies^23–25^. Tumor ploidy profiles estimated from WES data also suggest that polyploidization was prevailing in ACRC but not in PCRC (Supplementary Fig. 12). Similar to ITH of mutations, ITH of CNAs was observed in both PCRC and ACRC (Fig. 4a, Supplementary Figs. 13 and 14). By focusing on chromosomal arm-level CNAs, we compared the distributions of ubiquitous and heterogeneous CNAs between PCRC and ACRC. Overall, ACRC acquired significantly more ubiquitous CNAs than PCRC, while the numbers of heterogeneous CNAs were not significantly different (see Methods; Figs. 4b–d; *P* = 0.047 and 0.16, respectively; Wilcoxon rank-sum test). It should be noted that all samples were carcinoma in PCRC05, which harbored the maximum number of ubiquitous CNAs among PCRCs. By contrast, ACRC7, the ACRC case with a heterogeneous *TP53* mutation, harbored no ubiquitous CNAs; heterogeneous CNAs were observed only in samples with the *TP53* mutation (Supplementary Figs. 2, 5, and 13). We also found that ACRCs harbored an increased number of ubiquitous alterations for some of the chromosomal arms that are recurrently altered in the CRC population^19,23–26^. Such CNAs contain 20q amplification, which is established as a driver event for CRC progression^26–29^ (Fig. 4e). Collectively, our data suggest that CNAs act as a driver and are subject to selective sweep during progression from PCRC to ACRC.

## Discussion

In this study, we thoroughly characterized and compared ITH in PCRC and ACRC. Although some studies^30–32^ have examined ITH of PCRC, no conclusive view has been established. Similar to ITH in ACRC^13^, our multiregion sequencing unveiled extensive ITH in PCRC. In contrast to the neutral evolution model previously proposed for ACRC^13^, multiple lines of evidence indicate that at least a part of ITH of PCRC is shaped by Darwinian evolution. We found that multiple PCRC cases have evolutionary trees of forked tree-like shapes, which were not observed for ACRC. More direct evidence of Darwinian evolution was the observation that a significantly higher proportion of driver mutations accumulated as heterogeneous mutations in PCRC than in ACRC. (For simplicity, we discuss all results of the ubiquitous-heterogeneous categorization since analyses of the trunk-branch categorization also produced similar results.) In particular, heterogeneous mutations in *APC* and *KRAS* were noteworthy; these mutations were completely recognized as ubiquitous events in ACRC^13,14^. Our observation is perfectly supported by a prescient study that focused on only well-known driver mutations and LOH and reported that early colorectal tumors harbored more subclonal alterations than advanced tumors^33^. We also demonstrated that VAFs of shared mutations were higher in PCRC, additionally supporting the Darwinian evolution model^22^. We found that the number of somatic mutations per sample did not differ much between PCRC and ACRC. Although this finding may appear to contradict the fact that cancer genomes progressively accumulate mutations, it can be explained by the higher VAFs of PCRC subclonal mutations, which increased the sensitivity of mutation detection by WES. We also found that PCRC tended to harbor fewer ubiquitous mutations and more heterogeneous mutations than ACRC. However, the small cohort size limited the power of the statistical analysis (the ten PCRC cases vs. the eight ACRC cases); larger cohort size is necessary to confirm this tendency.

As for CNAs, their number progressively increased from adenoma through early carcinoma to ACRC; the increase in ubiquitous CNAs was especially prominent in ACRC. Together with the observation that mutations in well-known driver genes were already present in PCRC, these findings suggest that CNAs play more critical roles in the progression from PCRC to ACRC. This view is consistent with a recent report that CRISPR–Cas9-mediated engineering of canonical driver genes was not sufficient to confer invasive capacity to human intestinal organoids; the report also found that a chromosomal instability phenotype was necessary for metastatic behavior^34^. A number of recent studies have demonstrated that genome-wide mutational events such as whole-genome duplication (WGD)^4,7,35,36^ and chromothripsis^36^ play essential roles in tumor progression. Although our WES-based tumor ploidy profiling identified signatures of polyploidization in ACRC but not in PCRC, copy number analysis with a higher resolution is required to prove that WGD is involved in the progression from PCRC to ACRC. It is also possible that chromothripsis delineates a boundary between PCRC and ACRC; we should explore this possibility in future studies employing whole-genome sequencing.

**Fig. 5:**
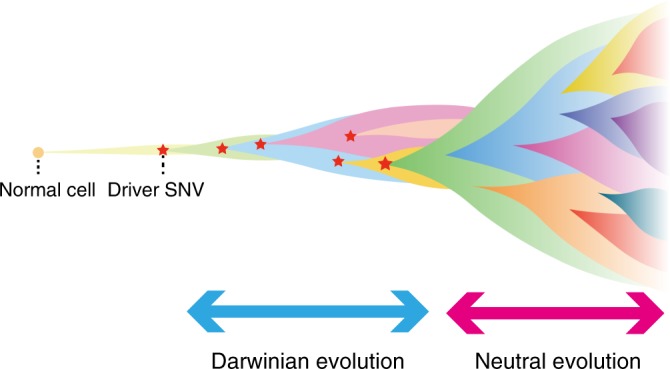
Our model of colorectal cancer evolution. During early tumorigenesis, multiple subclones harboring heterogeneous mutations on different driver genes appear and constitute ITH by Darwinian evolution. The tumor is then confronted with growth limitation before progressing to the late phase of tumorigenesis. Out of the multiple subclones generated by Darwinian evolution, the parental clone that can conquer the growth limitation emerges. In addition to a sufficient set of driver single-nucleotide mutations, such a clone possibly acquires driver CNAs. The parental clone is selected to progress locally advanced cancer or metastatic cancer. During the late phase, extensive ITH is generated by neutral evolution

Finally, we propose a model of CRC evolution that can simply explain our data (Fig. 5). In our model, multiple subclones are generated by driver mutation acquisition and subsequent selective sweep in PCRC because early tumor growth is inevitably hampered by obstacles such as spatial and nutritional limitation^37^ and immune attack^38^. However, out of the multiple subclones generated by Darwinian evolution, the parental clone that can conquer the obstacles emerges. In addition to a sufficient set of driver mutations, such a clone possibly acquires driver CNAs that endow a tumor with malignant phenotypes such as invasion, angiogenesis, and immune escape, and then it dominantly regrows by overcoming the obstacles. This evolutionary bottleneck establishes all driver mutations composing the parental clone in PCRC as ubiquitous mutations in ACRC, and the parental clone then branches into numerous subclones by neutral evolution. This model is consistent with the well-established multi-step carcinogenesis model of CRC^12^, in which mutations in major driver genes such as *APC, KRAS*, and *TP53* are sequentially accumulated in adenoma and then additional CNAs are acquired during the progression from adenoma to carcinoma. The neutral evolution phase in our model is also consistent with the recently proposed Big Bang model^14^, where a tumor predominantly grows as a single expansion without selective sweep. The extensive ITH generated by neutral evolution definitively works as a rich source of therapy-resistant subclones. However, considering recent reports that certain subclones that have branched out from a primary tumor in the early evolutionary phase constitute recurrent lesions after chemotherapy or radiotherapy^39–42^, it is also possible that subclones that appear in PCRC but that were weeded out by the evolutionary bottleneck remain as minimal residual clones, eventually contributing to therapeutic resistance. Further sequencing studies targeting recurrent lesions are necessary to elucidate more details of CRC evolutionary history.

For a long time after the establishment of the multi-step carcinogenesis model^12^, CRC was assumed to be a clonal cell population originating from linear clonal evolution. However, this view has recently been revised by a series of studies proposing that neutral evolution shapes extensive ITH in ACRC^13,14,17^. As an extension of these studies, this study provides a detailed view of ITH in PCRC and demonstrates that the evolutionary principle shaping ITH shifts from Darwinian to neutral evolution during CRC progression. We believe that our model of CRC evolution not only provides deep insights into the origin of ITH but also constitutes a foundation for conquering this malignancy.

## Methods

### Ethics statement

The study design was approved by the institutional review boards and ethics committees of the patients’ hospitals (Oita University Hospital Institutional Review Board: Protocol Number P-14-09, Kyushu University Institutional Review Board: Protocol Number 595-01). The study was conducted according to the principles expressed in the Declaration of Helsinki. We obtained written informed consent from all the patients in this study. There were no animal experiments in the study.

### Sample collection and preparation

We obtained 53 samples of colorectal tumors from ten patients with colorectal LST who underwent endoscopic submucosal dissection or radical resection at Kyushu University Beppu Hospital (Beppu, Japan) or Oita University Hospital (Yufu, Japan). These samples were histologically diagnosed by two qualified pathologists as adenoma or carcinoma in situ. Cancer cells were only found in the epithelium or lamina propria without vessel invasion. Following the 2010 WHO classification of tumors of the digestive system, the pathologists evaluated low- and high-grade dysplasia of each sample, which corresponded to adenoma and carcinoma in situ, respectively. Detailed information about participants and samples is provided in Supplementary Data 1. To use high-purity tumor samples, we performed microdissection of all frozen multiregion tumor samples using a Leica Laser Microdissection System (Leica Microsystems, Wetzlar, Germany), distinguishing between adenoma and carcinoma based on the diagnosis of the pathologists. However, when the volume of samples that consisted of both adenoma and early carcinoma was not sufficient, we captured only one of them. We included the diagnostic information in the sample names: the last character of the sample name, “A” or “C”, meant “adenoma” or “carcinoma”, respectively. DNA was extracted from these captured tumor samples and adjacent normal intestinal mucosa with AllPrep DNA/RNA Mini Kit (Qiagen, Hiden, Germany)^43^.

### Whole-exome sequencing

Whole-exome capture was performed on all PCRC samples with the SureSelect Human All Exon V5 Kit (Agilent Technologies, Tokyo, Japan). The captured targets were subjected to sequencing using HiSeq 2500 (Illumina, San Diego, CA, USA) with the pair-end 100 bp read option. Information on read depth is provided in Supplementary Data 2. The sequence data were processed through an in-house pipeline^44^. Briefly, the sequencing reads were aligned to the NCBI Human Reference Genome Build 37 hg19 with BWA version 0.7.8 using default parameters (http://bio-bwa.sourceforge.net/). PCR duplicate reads were removed with Picard (http://www.picard.sourceforge.net). Mutation calling was performed using the EBcall algorithm^45^ with the following parameters: (i) mapping quality score ≥ 20, (ii) base quality score ≥ 15, (iii) both the tumor and normal depths ≥ 8, (iv) variant reads in tumors ≥ 4, (v) VAF in tumor samples ≥ 0.05, (vi) VAF in paired normal samples ≤ 0.1, (vii) minus logarithm of *p* value of Fisher’s exact test ≥ 1.3, and (viii) minus logarithm of *p* value of EBcall ≥ 5. The filtered mutations were annotated by ANNOVAR ver.2015Dec14 (http://www.openbioinformatics.org/annovar/). As for ACRC, the WES data obtained in our previous study^13^ were reanalyzed by the same pipeline as for PCRC.

### Analysis of multiregion mutation profiles

For each case, we first obtained variants satisfying both the following criteria: (i) it was judged as a somatic mutation by EBcall in any sample and (ii) its position was covered by more than ten reads in all the samples. For each of the passed variants, we reexamined the presence of somatic mutations in each of the samples where EBcall did not judge the variant as a somatic mutation. In this step, which aimed to rescue false negatives missed by EBcall, we assumed the variant to be a somatic mutation if the variant satisfied all the following criteria: (i) VAF in the tumor sample ≥ 0.05, (ii) VAF ≤ 0.01 in the paired normal sample, and (iii) *p* value of Fisher’s exact test ≤ 0.05. This procedure was applied to each case to obtain a multiregion mutation profile, and then we defined mutations shared by all the samples in each case and other mutations as ubiquitous mutations and heterogeneous mutations, respectively. Heterogeneous mutations were further divided into shared mutations, which were shared by multiple samples, and private mutations, which uniquely existed in a single sample. Information for all the mutations is provided in Supplementary Data 3. The multiregion mutation profile obtained for each case was visualized as a heat map, in which intensities represented VAFs. In the heat map, ubiquitous mutations were ordered along chromosomal positions; shared mutations were ordered by a hierarchical clustering; private mutations were sorted for samples and VAFs. The list of the driver genes indicated in the heat maps was based on the significantly mutated genes that had been previously reported for CRC^18^ (Supplementary Table 1). Colors of PCRC sample labels were obtained in the same way as in our previous study^13^. Namely, from the multiregional mutation profile of each case, we also deduced a color-coding scheme to prepare color labels of samples. The multiregional mutation profile were regarded as an n × m matrix, whose n columns and m rows indexed n mutational positions and m samples, respectively. We applied principle component analysis to the multiregional mutation profile and obtained the first, second and third loading vectors. By multiplying these loading vectors, *n*-dimensional vectors representing mutational profiles of each sample were reduced into three-dimensional vectors. RGB colors used for sample labels are finally papered by mixing red, green, and blue proportionally to the three vector elements. In a color-coding scheme deduced by this approach, color similarity reflects similarity of mutation profiles between samples. For ACRC samples, we employed the same colors as used in our previous study^13^.

### Mutation validation by targeted deep sequencing

We performed amplocon-sequencing of tumor DNA for 73 candidate mutations chosen randomly from ubiquitous, shared, and private mutations, based on a previously reported protocol^46^. Briefly, regions containing candidate mutations were amplified from 10 ng of DNA using KOD plus neo (TOYOBO, Osaka, Japan) with primers that were attached by NotI sequences at the 5′ end. Successful amplification was confirmed by gel electrophoresis. Amplicons were pooled, purified using the FastGene Gel/PCR 5 Extraction Kit (Nippon Genetics, Tokyo, Japan), and digested with NotI restriction enzyme (Takara Bio, Shiga, Japan), according to the instruction manual. Samples were ligated using T4 DNA polymerase (Takara Bio) and re-purified; ligated DNA was sonicated into ~200 bp fragments using a Covaris sonicator (Covaris inc., Massachusetts, USA), and prepared for generation of sequencing libraries using NEBNext Ultra DNA Library Prep Kit for Illumina (New England BioLabs, Massachusetts, USA). Libraries were then subjected to deep sequencing on a Hiseq 2500 instrument. Candidate mutations were considered real if both of the following criteria were satisfied: (i) VAF in the tumor ≥ 0.01 and (ii) sequencing depth ≥ 500.

### Construction of evolutionary trees

From the multiregion sequencing data for each case, an evolutionary tree was constructed using the Treeomics algorithm^21^ (https://github.com/johannesreiter/treeomics) with default parameters. For every mutation existing in the multiregion mutation profile, the numbers of variant reads, read depth, chromosomal coordinates, gene symbol, and substitution pattern were prepared as input data to Treeomics. Treeomics not only constructs an evolutionarily tree but also corrects potential sequencing artifacts so that all mutations have mutation patterns compatible with the topologies of the evolutionary tree. Based on the parts of the tree that the mutations constituted, we obtained trunk, branch, internal branch, and external branch mutations, which were refined versions of ubiquitous, heterogeneous, shared and private mutations, respectively. To remove potential sequencing artifacts, Treeomics also employs mutation filters, which filtered out 1.3% of our input mutations. Information about the trunk-branch categorization is also provided in Supplementary Data 3. We were unable to apply Treeomics to the ACRC3 data, which contained 21 samples, due to insufficient memory on our computer. To address this problem, we divided the ACRC3 data into two parts which corresponded to two apparent sample clusters in the multiregion profiles. After the divided data were subjected to Treeomics, an evolutionary tree was constructed by merging the results. Except for ACRC2 and ACRC3, the robustness of the evolutionary tree inference was examined on 1000 bootstrapping samples from the input mutations. For ACRC2 and ACRC3, only 50 bootstrapping samples were obtained due to the memory limitation. The inferred evolutionary trees were annotated with the same driver gene list as used for the heat maps of the multiregion mutation profiles (Supplementary Table 1). For detection of subclonal mixing, we reconstructed evolutionary trees with the “-u” option and the obtained information of subclonal mixing was added to the trees constructed without the “-u” option (Supplementary Figs. 6 and 7).

### Analysis of CNAs

To detect CNAs from WES data, we used a software tool, EXCAVATOR^47^ (http://sourceforge.net/projects/excavatortool/), that not only reports chromosomal segments subjected to CNAs but also outputs the log-transformed ratio of copy number intensities between tumor and normal samples (log_2_R) for each locus. We used twice the median of the ubiquitous mutation allele frequencies for each sample as the cellularity parameters. CNAs whose length was larger than 50% of the chromosomal arm were classified as chromosomal arm-level CNAs, while the others were classified as focal CNAs. For each case, we made a multiregion arm-level CNA profile, which presented an average log_2_R for each of the chromosomal arms subjected to CNAs. For each of the chromosomal arms that EXCAVATOR reported to have CNAs in any sample, we reexamined a presence of CNAs in each sample where EXCAVATOR did not report the chromosomal arm-level CNA; we assumed that a CNA existed if the absolute value of log_2_R averaged along the chromosomal arm was greater than 0.15. We also prepared a multiregion focal CNA profile for each case, by focusing on candidate loci that were previously reported to be recurrently altered^19,26^. For each candidate locus that had overlap with EXCAVATOR-deduced focal CNAs in any sample, we calculated log_2_R averaged along the locus for each sample. We assumed that a CNA existed if the absolute value of the averaged log_2_R was greater than 0.15.

### Estimation of tumor ploidy

To estimate tumor ploidy from WES data, we used two software tools, FACETS^48^ (https://github.com/mskcc/facets) and sequenza^49^ (https://cran.r-project.org/web/packages/sequenza/index.html). In FACETS, we prepared germ line polymorphic sites cataloged in the Human Genetic Variation Database version 2.30 (http://www.hgvd.genome.med.kyoto-u.ac.jp) as a reference. Other parameters were set by default in both FACETS and sequenza.

### Comparison of the numbers of ubiquitous and heterogeneous alterations between cases

We reanalyzed our previously published multiregion WES data sets of ACRC^13^ in the same way that PCRC data were analyzed for calling mutations and CNAs. Although the data set contained nine ACRCs, one hypermutated case was excluded in this study. The prefix “case” in the previous sample names was also replaced with “ACRC”. Each of the ten PCRC and eight ACRC cases had a different number of samples from 4 to 21, which led to an unfair comparison of the numbers of ubiquitous and heterogeneous alterations. We addressed this problem by employing a down-sampling approach, where the numbers of ubiquitous and heterogeneous alterations were estimated from randomly sampled sub-datasets of an equal number of samples across all cases, using the following steps: (i) for each case, we obtained every sub-dataset with four samples, which was the minimum number of samples in our data set; (ii) for each sub-dataset, the numbers of ubiquitous and heterogeneous alterations were calculated (here, since heterogeneous alterations were associated with each sample, we took the median across samples as the number of the heterogeneous alterations); and (iii) medians across all sub-datasets were assumed to be corrected numbers of ubiquitous and heterogeneous alterations. An explanatory example of our down-sampling approach is provided in Supplementary Fig. 15.

### Comparison of the numbers of alterations between different tumor stages

To compare the number of mutations between different tumor stages, we employed hierarchical Bayesian analysis (Supplementary Fig. 16), which enabled us to estimate the mean number of mutations in each tumor stage, after removing the residuals associated with samples and cases in which the mutations were found. As the tumor stages, we assumed the following three categories: *T*^(SNV)^ = {adenoma, carcinoma, ACRC}. Let *i* and *j* denote the indices for samples and cases, respectively. From multiregion sequencing data of *I* cases, each of which contains *J*_*i*_ samples, we obtain the number of mutations in the *j*-th sample of the *i* case (hereafter, simply referred as to sample *ij*) as *n*_*ij*_^(SNV)^ (*i* = 1, …, *I* and *j* = 1, …, *J*_*i*_). Sample *ij* is associated with any of the three tumor stages: *t*_*ij*_ ∈ *T*^(SNV)^. We assume that *n*_*ij*_^(SNV)^ is sampled from a Poisson distribution: *n*_*ij*_^(SNV)^ ~ Poisson(*μ*_*ij*_^(SNV)^), where *μ*_*ij*_^(SNV)^ is expressed by the main term associated with tumor stages and the residual term associated with samples and cases: *μ*_*ij*_^(SNV)^ ~ exp(*β*_*ij*_^(SNV)^ + *r*_*ij*_^(SNV)^). The main term *β*_*ij*_^(SNV)^ is obtained by substituting the tumor stage of sample *ij*, *t*_*ij*_, into variable *β*_*t*_^(SNV)^, which represents the mean number of mutations in tumor stage *t* ∈ *T*^(SNV)^: *β*_*ij*_^(SNV)^ ← *β*_*tij*_^(SNV)^. To ensure the robustness of parameter estimation, we employed a Cauchy distribution as the prior distribution for *β*_*t*_^(SNV)^: *β*_*t*_^(SNV)^ ~ Cauchy(*β*^(SNV)^, *τ*^(SNV)^). The two hyper-parameters are also sampled from Cauchy and half-Cauchy^50^ hyper-priors, respectively: *β*^(SNV)^ ~ Cauchy(*β*_0_^(SNV)^, *τ*_0_^(SNV)^) and *τ*^(SNV)^ ~ Half-Cauchy(*λ*_0_^(SNV)^), where we set *β*_0_^(SNV)^ = 0, *τ*_0_^(SNV)^ = 1, and *λ*_0_^(SNV)^ = 1. The residual term *r*_*ij*_^(SNV)^ is hierarchically sampled from two Cauchy distributions: *r*_*i*_^(SNV)^ ~ Cauchy(*r*_0_^(SNV)^, *t*^(SNV)^) and *r*_*ij*_^(SNV)^ ~ Cauchy(*r*_*i*_^(SNV)^, *t*_*i*_^(SNV)^). We set *r*_0_^(SNV)^ = 1 while the scale parameters are sampled from half-Cauchy hyper-priors: *t*^(SNV)^ ~ Half-Cauchy(*l*_0_^(SNV)^) and *t*_*i*_^(SNV)^ ~ Half-Cauchy(*l*_1_^(SNV)^), where we set *l*_0_^(SNV)^ = 1 and *l*_1_^(SNV)^ = 1. For each of the 123 samples in the ten PCRCs and eight ACRCs, the number of all mutations and tumor stage was prepared as *n*_*ij*_^(SNV)^ and *t*_*ij*_, respectively. We estimated the posterior distribution of *β*_*t*_^(SNV)^ by running MCMC on JAGS 4.2.0^51^ with the following parameter settings: number of chains = 20, number of burn-in iterations = 100,000, number of total iterations = 200,000 and thinning interval = 5. Convergence of Markov chains was confirmed by the Gelman-Rubin convergence diagnostic^52^. The density plot in Supplementary Fig. 1c shows the distribution of the MCMC samples of *β*_*t*_^(SNV)^ for each tumor stage on the exponential scale. To compare of the number of CNAs, the numbers of CNAs in the 123 samples were prepared and processed in the same way, except for the MCMC parameter settings: number of chains = 20, number of burn-in iterations = 50,000, number of total iterations = 100,000 and thinning interval = 5 (Supplementary Fig. 11e).

### Comparison of VAFs between different categories of mutations

Hierarchical Bayesian analysis was employed to compare VAFs between different categories of mutations, similarly to the comparison of the numbers of alterations between different tumor stages (Supplementary Fig. 17). We estimated the mean VAFs for each category of mutations, after correcting for the effects of tumor content and read depth, as well as removing the residuals associated with individual mutations, samples and cases in which the mutations were found. We assume that mutations are categorized into the following six categories: *T*^(VAF)^ = {PCRC, ACRC} × {trunk, internal branch, external branch} (or {PCRC, ACRC} × {ubiquitous, shared, private} on the ubiquitous-heterogeneous categorization). In addition to the case index *i* and sample index *j*, let *k* denote the index for mutations. We assume that *K*_*ij*_ (=*n*_*ij*_^(SNV)^) mutations are identified in sample *ij* and the *k*-th mutation (hereafter, simply referred as to mutation *ijk*) is categorized as *t*_*ijk*_ (*k* = 1, …, *K*_*ij*_). For mutation *ijk*, we obtain the numbers of total and variant reads, which are represented as *d*_*ijk*_ and *b*_*ijk*_, respectively. We assume that *b*_*ijk*_ is sampled from the binomial distribution with parameters *d*_*ijk*_ (the number of trials) and *P*_*ijk*_^*(m)*^ (the success probability): *b*_*ijk*_ ~ Binomial(*d*_*ijk*_, *P*_*ijk*_^*(m)*^). *P*_*ijk*_^*(m)*^ is a modified VAF, which is obtained by multiplying the true VAF and the tumor content of sample *ij*: *P*_*ijk*_^*(m)*^ ← *P*_*ijk*_^*(t)*^ × *TC*_*ij*_. As with *μ*_*ij*_^(SNV)^, *P*_*ijk*_^*(t)*^ is expressed by the main term associated with mutation categories and the residual terms associated with individual mutations, samples and cases: *P*_*ijk*_^*(t)*^ ~ logistic(*β*_*ijk*_^(VAF)^ + *r*_*ijk*_^(VAF)^). The main term *β*_*ijk*_^(VAF)^ is obtained by substituting the category of mutation *ijk*, *t*_*ijk*_, into variable *β*_*t*_^(VAF)^, which represents the mean VAF of mutations of category *t* ∈ *T*^(VAF)^: *β*_*ijk*_^(VAF)^ ← *β*_*tijk*_^(VAF)^. *β*_*t*_^(VAF)^ is hierarchically sampled from Cauchy and half-Cauchy distributions in the same way as *β*_*t*_^(SNV)^. The residual term *r*_*ijk*_^(VAF)^ is sampled from a Cauchy distribution: *r*_*ijk*_^(VAF)^ ~ Cauchy(*r*_*ij*_^(VAF)^, *t*_*ij*_^(VAF)^). *r*_*ij*_^(VAF)^ is obtained in the same way as *r*_*ij*_^(SNV)^, while *t*_*ij*_^(VAF)^ is sampled from a half-Cauchy hyper-prior: *t*_*ij*_^(VAF)^ ~ Half-Cauchy(*l*_*2*_^(VAF)^), where we set *l*_*2*_^(VAF)^ = 1. For each of the mutations found in the 123 samples of the 10 PCRCs and 8 ACRCs, the numbers of total and variant reads and the mutation categories were prepared as *d*_*ijk*_, *b*_*ijk*_ and *t*_*ijk*_. For each sample, the tumor content estimated by Treeomics was used as *TC*_*ij*_. The posterior distribution of *β*_*t*_^(VAF)^ was estimated by MCMC on JAGS with the following parameter settings: number of chains = 20, number of burn-in iterations = 50,000, number of total iterations = 50,000 and thinning interval = 5. The density plot in Fig. 3c and Supplementary Fig. 9c show the distribution of the MCMC samples of *β*_*t*_^(VAF)^ for each mutation category after logistic conversion.

### Comparison of CCFs between different categories of mutations

To compare CCFs between different categories of mutations, we used a hierarchical Bayesian model similar to the one for the comparison of VAFs (Supplementary Fig. 18). Instead of *P*_*ijk*_^*(t)*^and *P*_*ijk*_^*(m)*^, we introduced *C*_*ijk*_ and *P*_*ijk*_, which represent CCF and VAF, respectively. We also added *CN*_*ijk*_, which represents the absolution copy number of the locus where mutation *ijk* exists. VAF is then represented as follows; *P*_*ijk*_  ← *TC*_*ij*_•*C*_*ijk*_/{(1−*TC*_ij_)•2 + *TC*_ij_•*CN*_ijk_}^53^. Except for these points, the CCF model is the same as the VAF model. As input data, we prepared absolute copy numbers estimated by EXCAVATOR in addition to the input data used for the VAF model. Mutations on sex chromosomes were removed from the input to the CCF analysis. The posterior distribution of *β*_*t*_^(CCF)^ was estimated by MCMC on JAGS with the following parameter settings: number of chains = 20, number of burn-in iterations = 200,000, number of total iterations = 200,000 and thinning interval = 5. The density plot in Supplementary Fig. 10 shows the distribution of the MCMC samples of *β*_*t*_^(CCF)^ for each mutation category after logistic conversion.

### Data availability

All WES data have been deposited in the Japanese Genotype-phenotype Archive with accession number JGAS00000000092 [https://humandbs.biosciencedbc.jp/en/hum0095-v1].

## Electronic supplementary material

Supplementary Information

Description of Additional Supplementary Files

Supplementary Data 1

Supplementary Data 2

Supplementary Data 3
