# Supplementary material for: A temporal shift of the evolutionary principle shaping intratumor heterogeneity in colorectal cancer
Source: Nat Commun. 2018 Jul 23;9:2884. doi: 10.1038/s41467-018-05226-0 (PMC6056524; doi:10.1038/s41467-018-05226-0)
Supplement: Supplementary file 1 — Supplementary Information [file 41467_2018_5226_MOESM1_ESM.pdf]

Supplementary Information

A temporal shift of the evolutionary principle shaping intratumor heterogeneity in colorectal cancer

Saito et al.

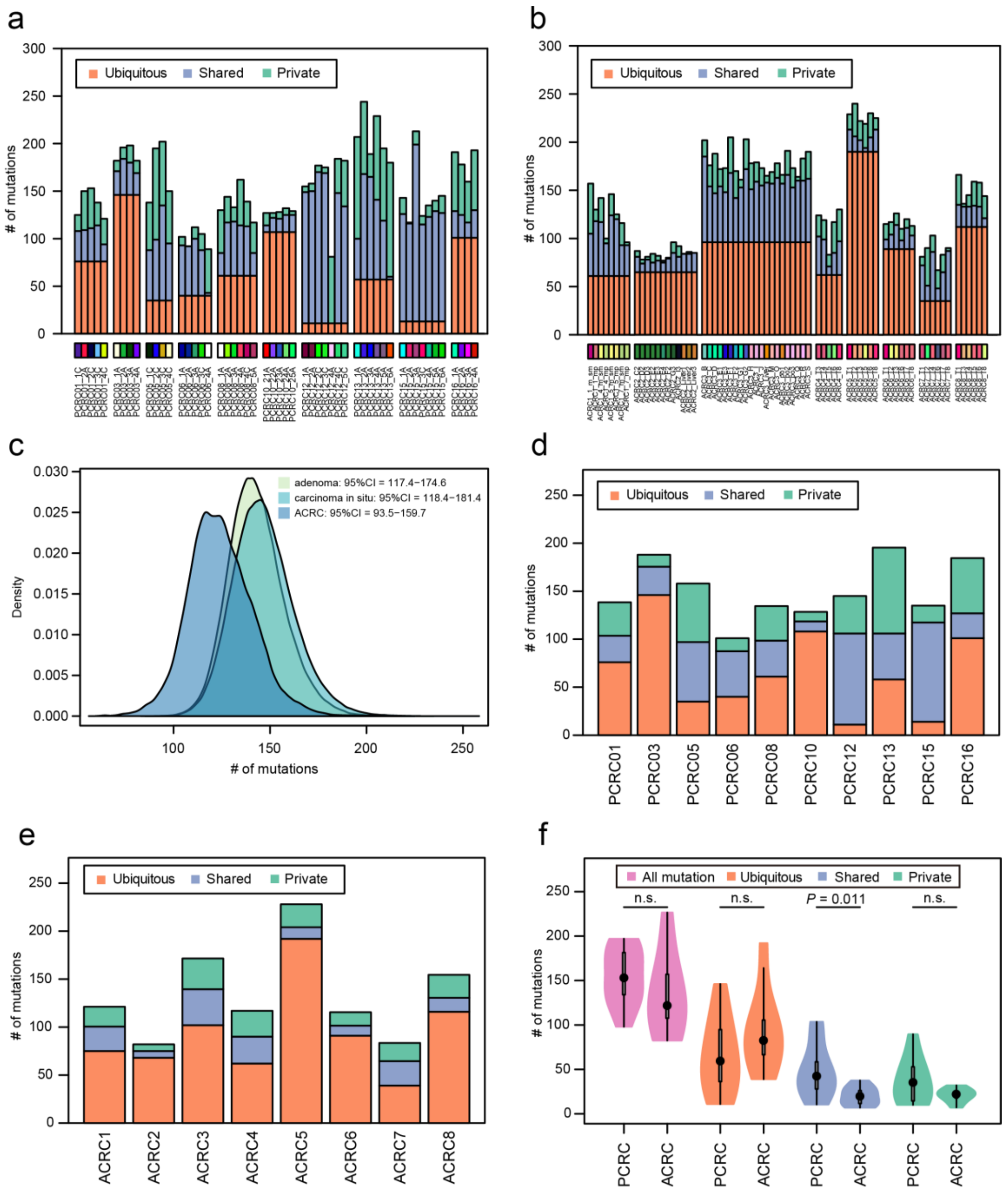

Supplementary Figure 1 Analysis of the number of somatic mutations. (**a**, **b**) Bar graphs showing the number of ubiquitous, shared, and private mutations identified by our WES for each sample in the 10 PCRC cases (a) and 8 ACRC cases (b). (**c**) Comparison the number of mutations between three different stages: adenoma (n = 38) and carcinoma (n = 15) in PCRC, and ACRC (n = 70). Hierarchical Bayesian analysis was employed to remove the residuals associated with samples and cases (see Methods). The density plot represents an estimated posterior distribution of the corrected mean numbers of mutations in adenoma, carcinoma in situ and ACRC. There were no differences between adenoma and carcinoma in situ, between carcinoma in situ and ACRC, and between adenoma and ACRC. (**d**, **e**) Bar graphs showing the number of ubiquitous, shared, and private mutations for each sample in the 10 PCRCs (d) and 8 ACRCs (e). Effects of the different numbers of samples between cases were corrected by down-sampling (see Methods). (**f**) Comparison of the numbers of all, ubiquitous, shared and private mutations between PCRC and ACRC. The violin plot represents the distribution of the numbers of each category of mutations. PCRC had more shared mutations than ACRC (*P* = 0.011; Wilcoxon rank-sum test). The distributions were obtained by correcting the counts that were presented for each case in (d) and (e). n.s., not significant; 95% CI, 95 percent credible interval.

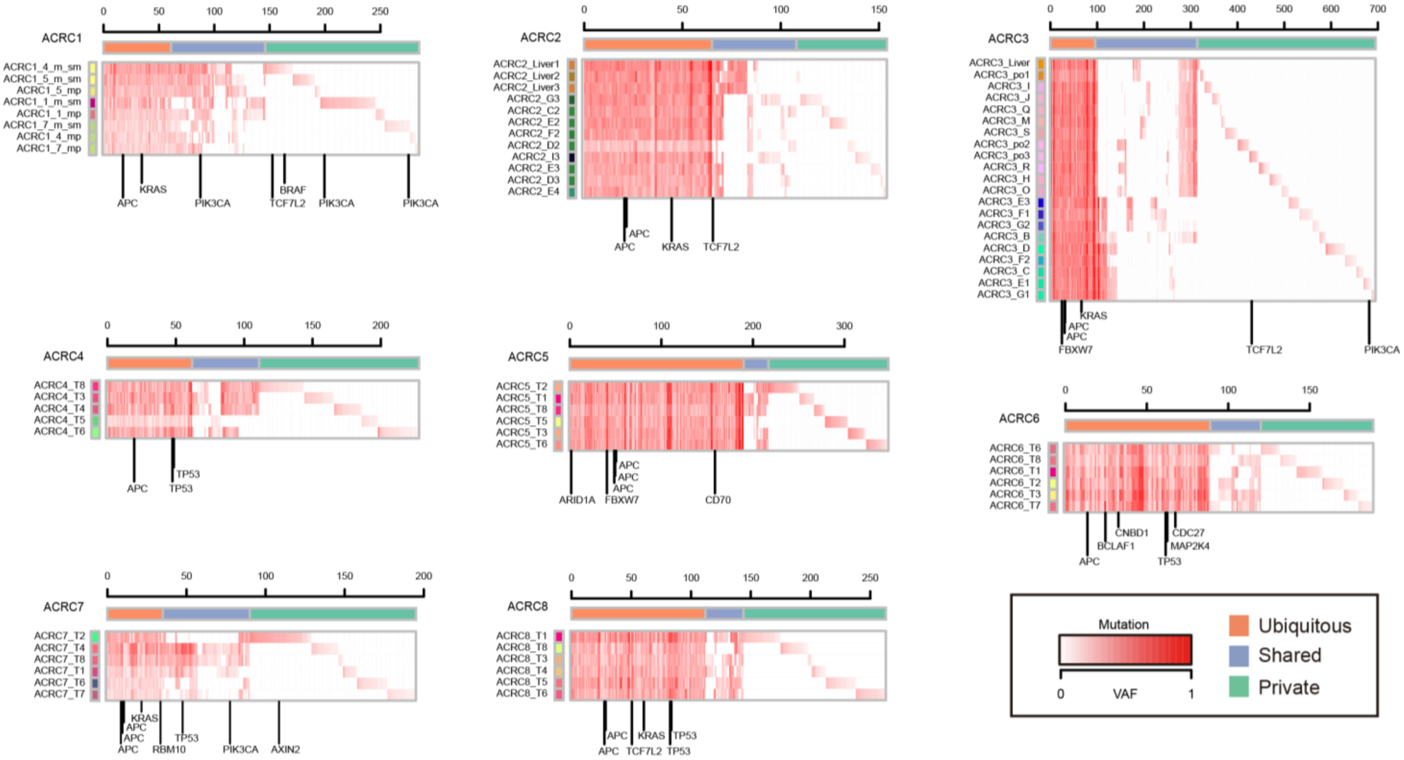

Supplementary Figure 2 Multiregion mutation profiles of ACRCs, presented as in **Fig. 1**. The colors of sample labels are the same as in our previous study (ref 13).

13. Uchi, R. et al. Integrated Multiregional Analysis Proposing a New Model of Colorectal Cancer Evolution. PLoS Genet. 12, e1005778 (2016).

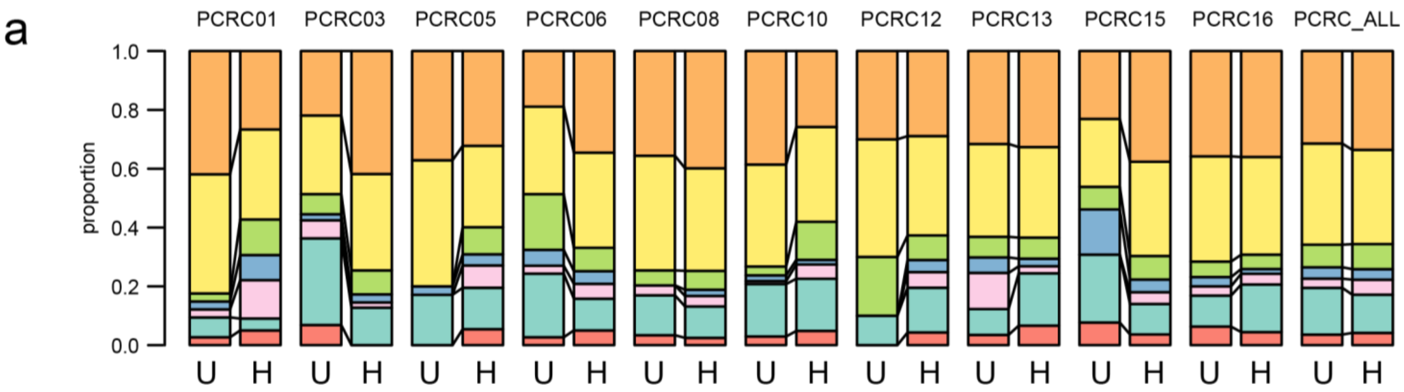

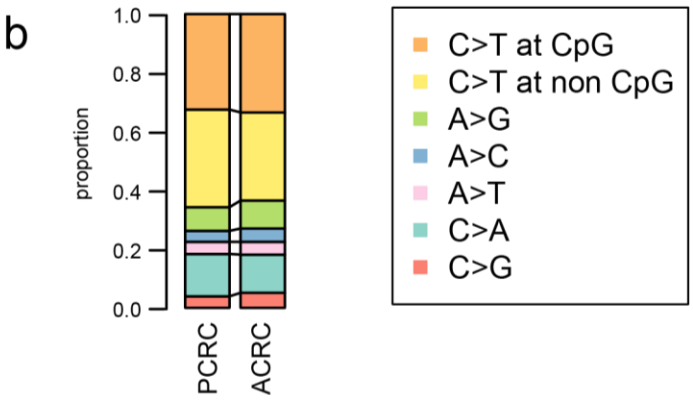

Supplementary Figure 3 Mutation spectra analysis. (**a, b**) Mutational spectra compared between ubiquitous (U) and heterogeneous (H) mutations in the 10 PCRC cases (a), and between PCRC and ACRC (b). No significant differences were observed by Wilcoxon signed-rank test (a) and Fisher's exact test (b).

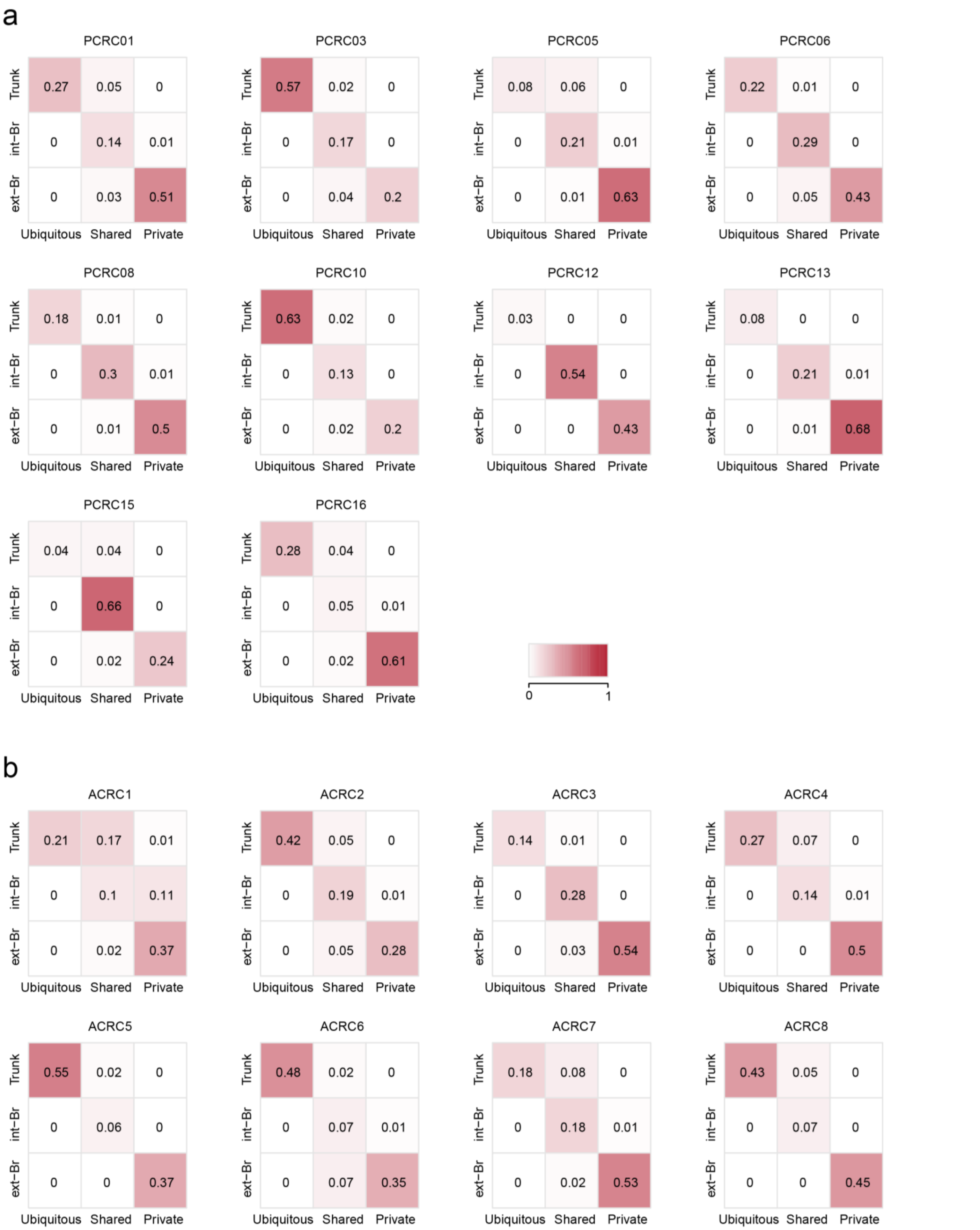

Supplementary Figure 4 Mapping of mutations between the ubiquitous-heterogeneous and trunk-branch categorizations. (**a**, **b**) For each sample of PCRC (a) and ACRC (b), each cell in the colored table indicates the proportion of mutations assigned to two categories on the ubiquitous-heterogeneous (horizontal-axis) and trunk-branch categorizations (vertical-axis). int-Br, internal branch; ext-Br, external branch.

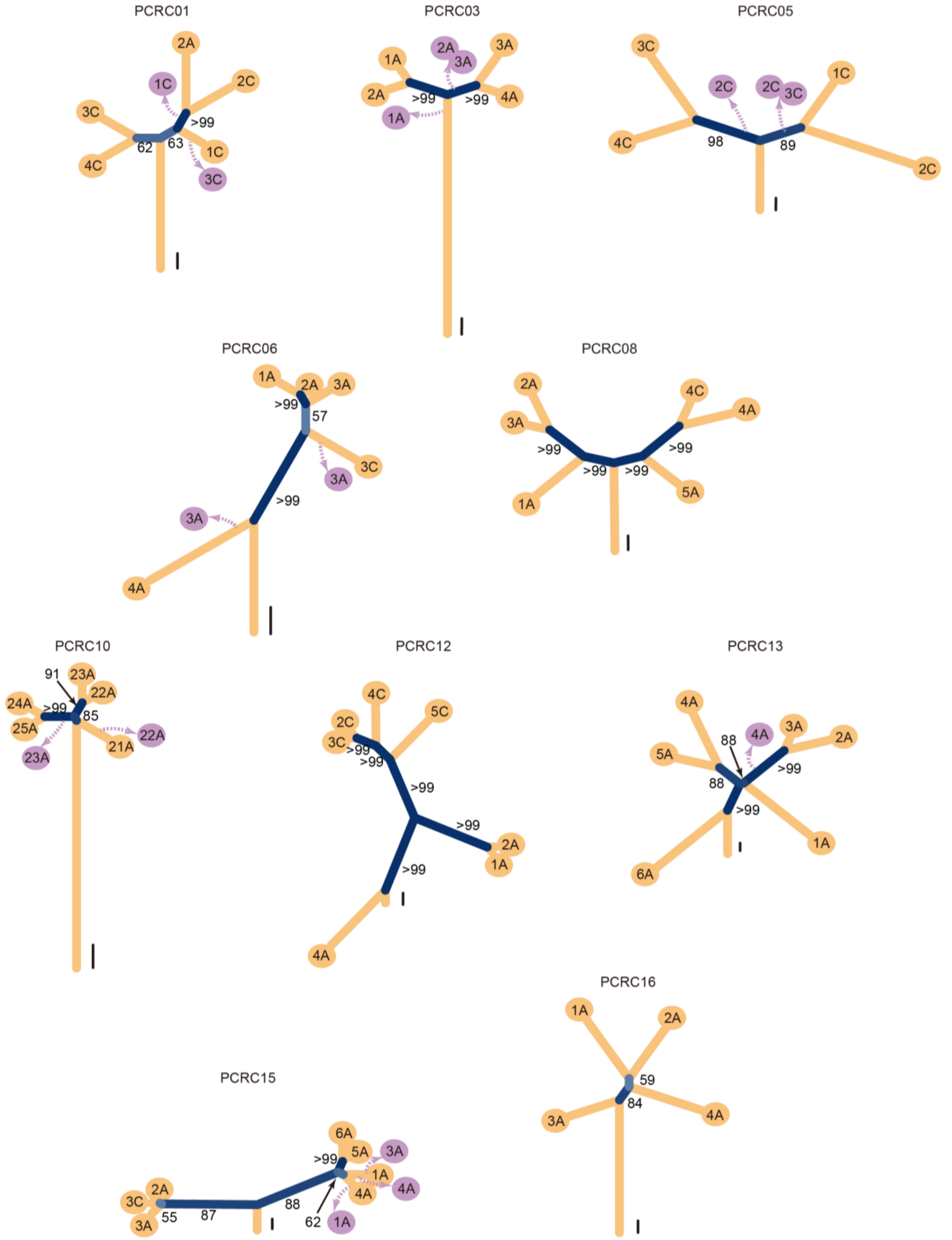

Supplementary Figure 5 Bootstrapping analysis and subclonal mixture detection on the PCRC evolutionary trees. The robustness of the evolutionary tree inference was examined by bootstrapping analysis in Treeomics. The depth of indigo on internal branches and associated digits represent bootstrapping values (percentages), i.e., how often the same branch was observed in bootstrap trials. Purple dashed arrows and sample labels represent subclonal mixture detected by Treeomics; i.e., a minor subclone originating from the branch associated with the arrow tail was mixed into the sample associated with the arrow head. Black lines near the roots of the trees represent scales for 10 mutations.

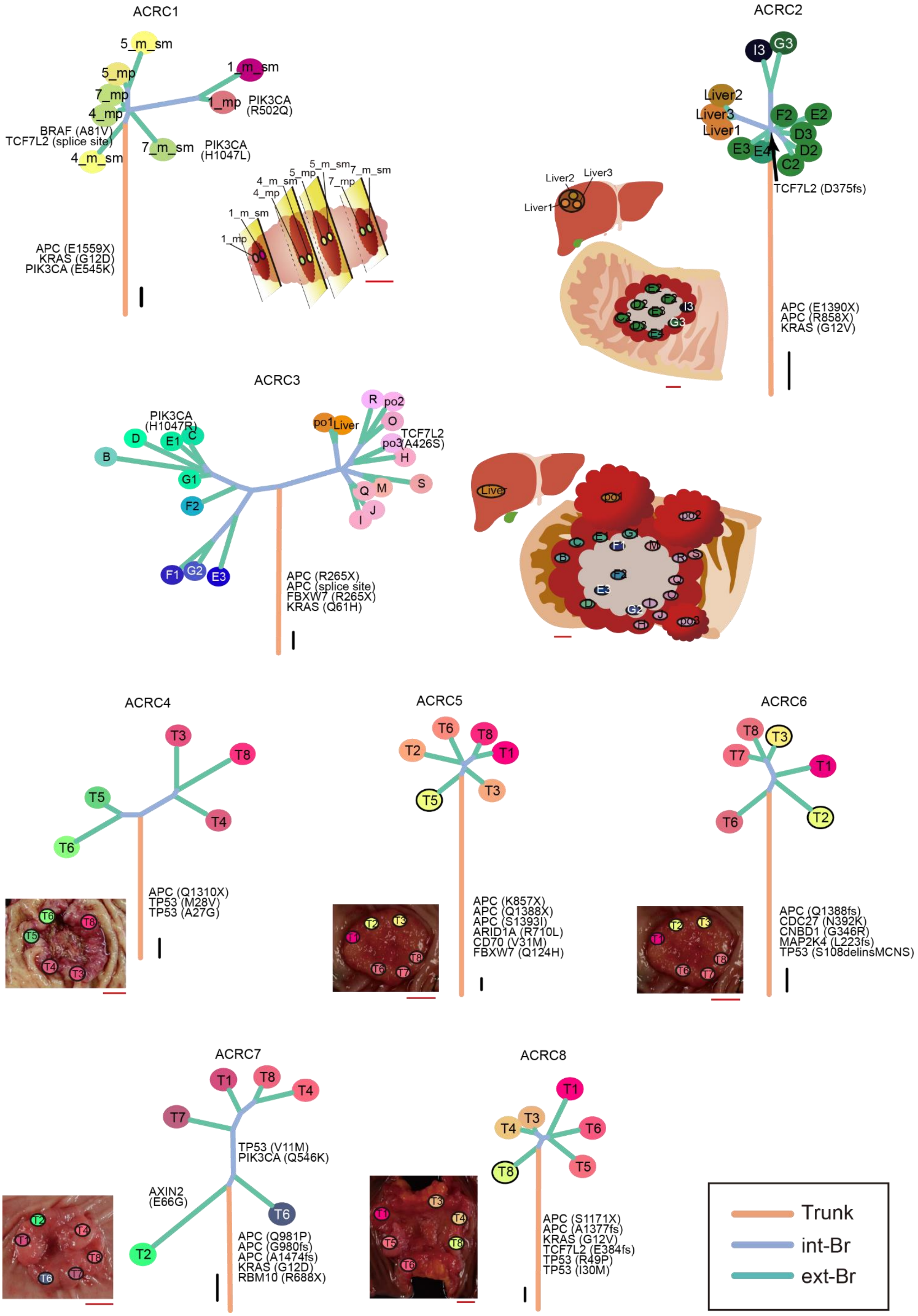

Supplementary Figure 6 Evolutionary trees of ACRCs, presented as in **Fig. 2**.

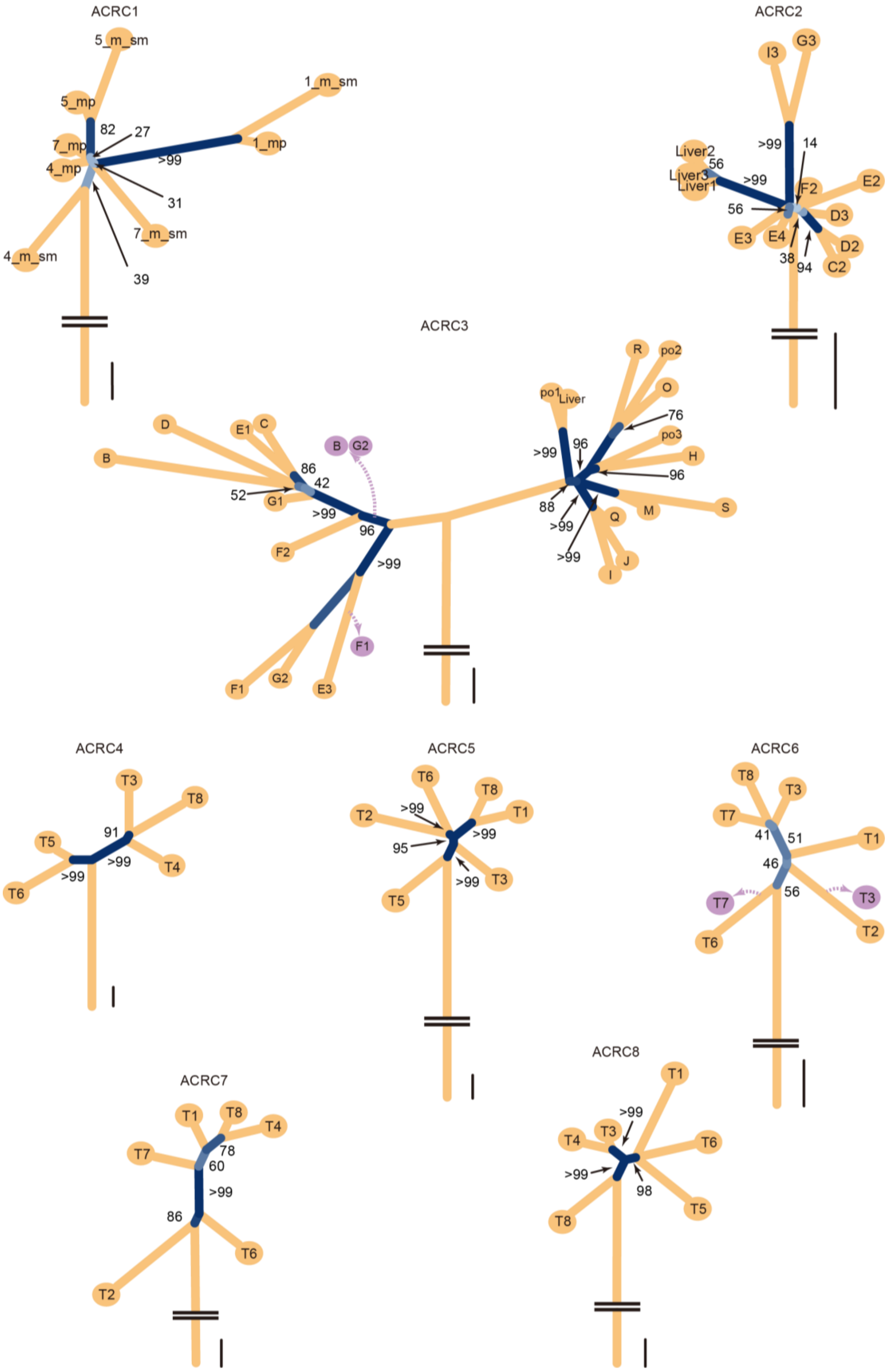

Supplementary Figure 7 Bootstrapping analysis and subclonal mixture detection on the ACRC evolutionary trees, presented as in **Supplementary Fig. 5**. In this figure, Horizontal double lines on the trunks indicate that the trunks were shortened.

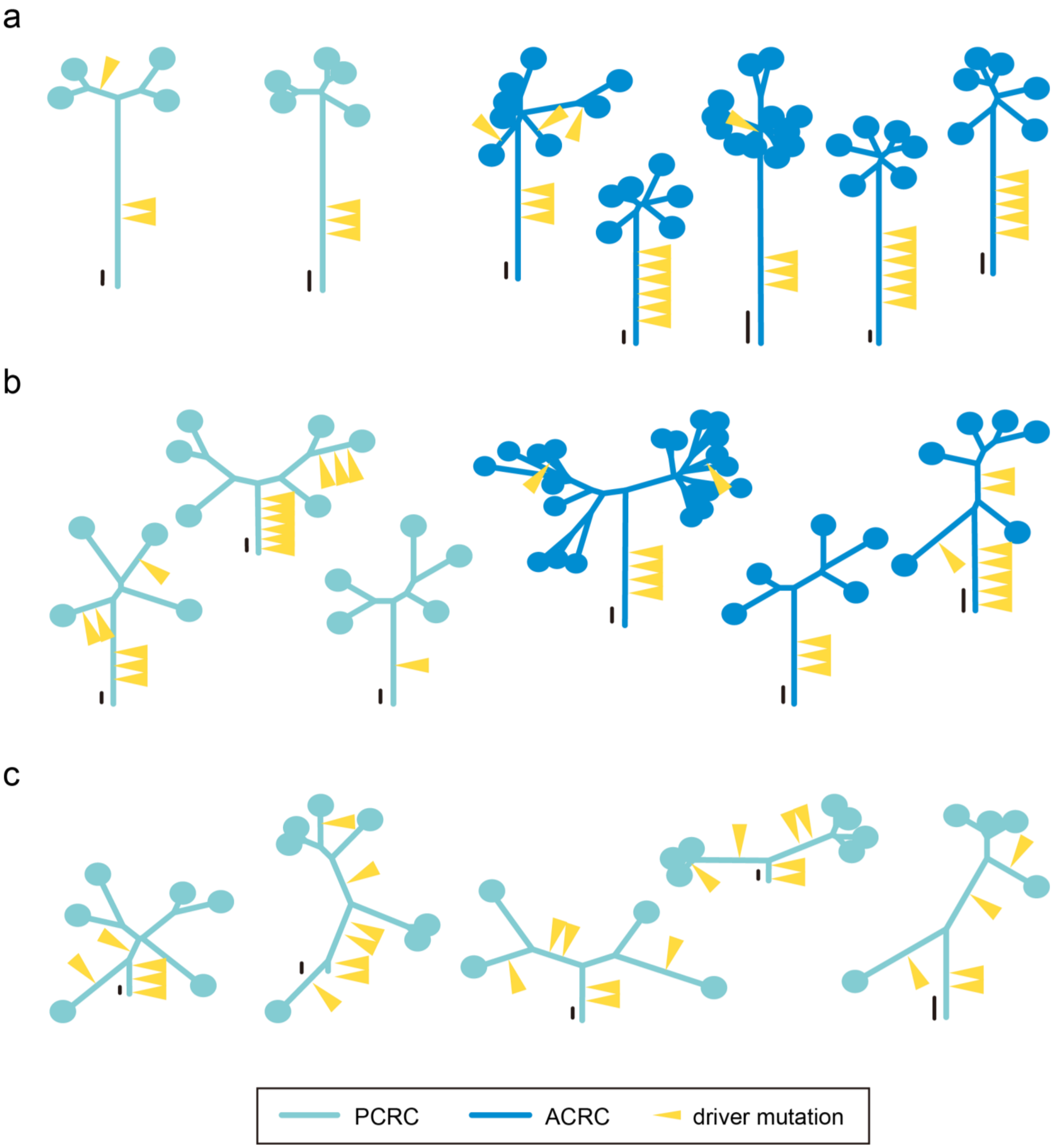

Supplementary Figure 8 Comparison of evolutionary trees between PCRC and ACRC. (**a**) Palm tree-like evolutionary trees. The length of trunk is long and the length of branches is short. (**b**) Intermediate type of evolutionary trees. (**c**) Forked tree-like evolutionary trees. The length of trunk is short and the length of branches is long. Light green and blue trees represent trees for the PCRC and ACRC cases, respectively. Yellow triangles denote driver mutations while black lines near the roots of the trees represent scales for 10 mutations.

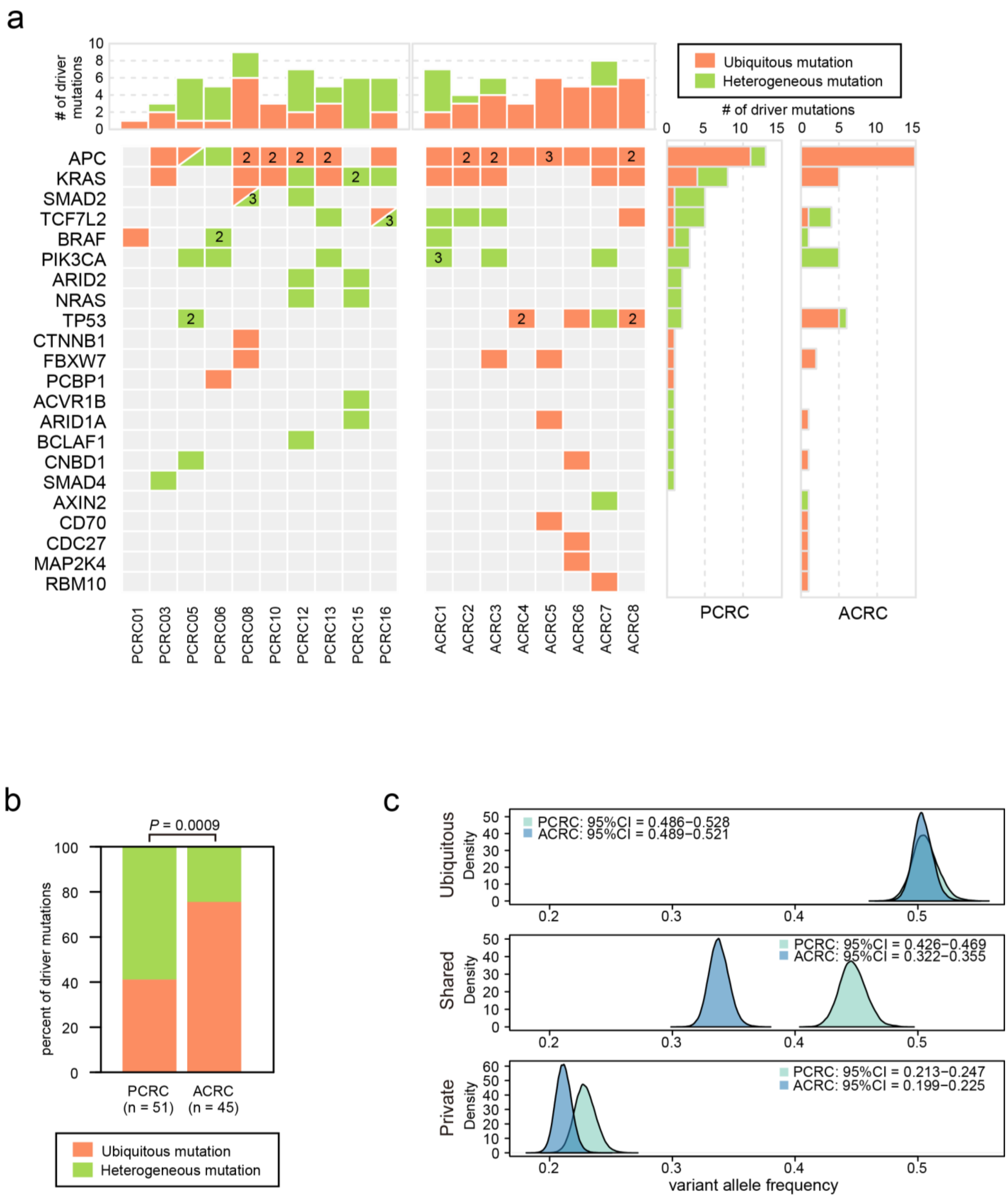

Supplementary Figure 9 Darwinian evolution mainly shapes ITH in PCRC. Analyses were performed based on the ubiquitous-heterogeneous categorization, in a similar manner to those in **Fig. 3**. (**a**) Distribution of driver genes. (**b**) Significant enrichment of heterogeneous mutations of driver genes in PCRC (30/51) was compared with ACRC (11/45; P = 0.00090; Fisher's exact test). (**c**) Comparison of VAFs for ubiquitous, shared and private mutations.

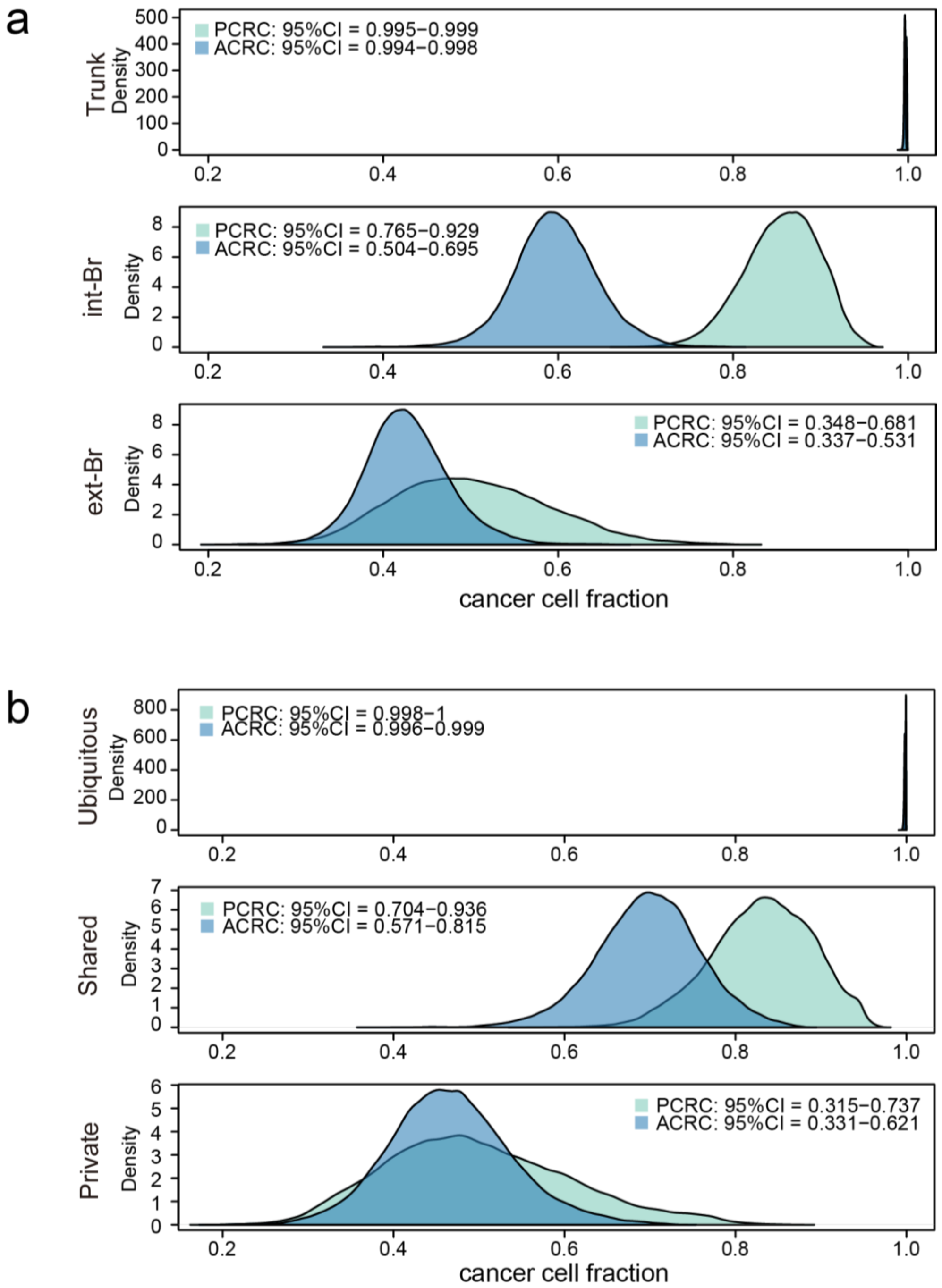

Supplementary Figure 10 Comparison of CCFs for different mutation categories. (**a**) CCFs for trunk mutations, internal branch (int-Br) mutations, and external branch (ext-Br) mutations. The density plot shows an estimated posterior distribution of mean CCFs, which was obtained by Hierarchical Bayesian analysis, similarly to the VAF analysis in **Fig. 3c** (see Methods). (**b**) CCFs for ubiquitous, shared and private mutations. 95% CI, 95 percent credible interval.

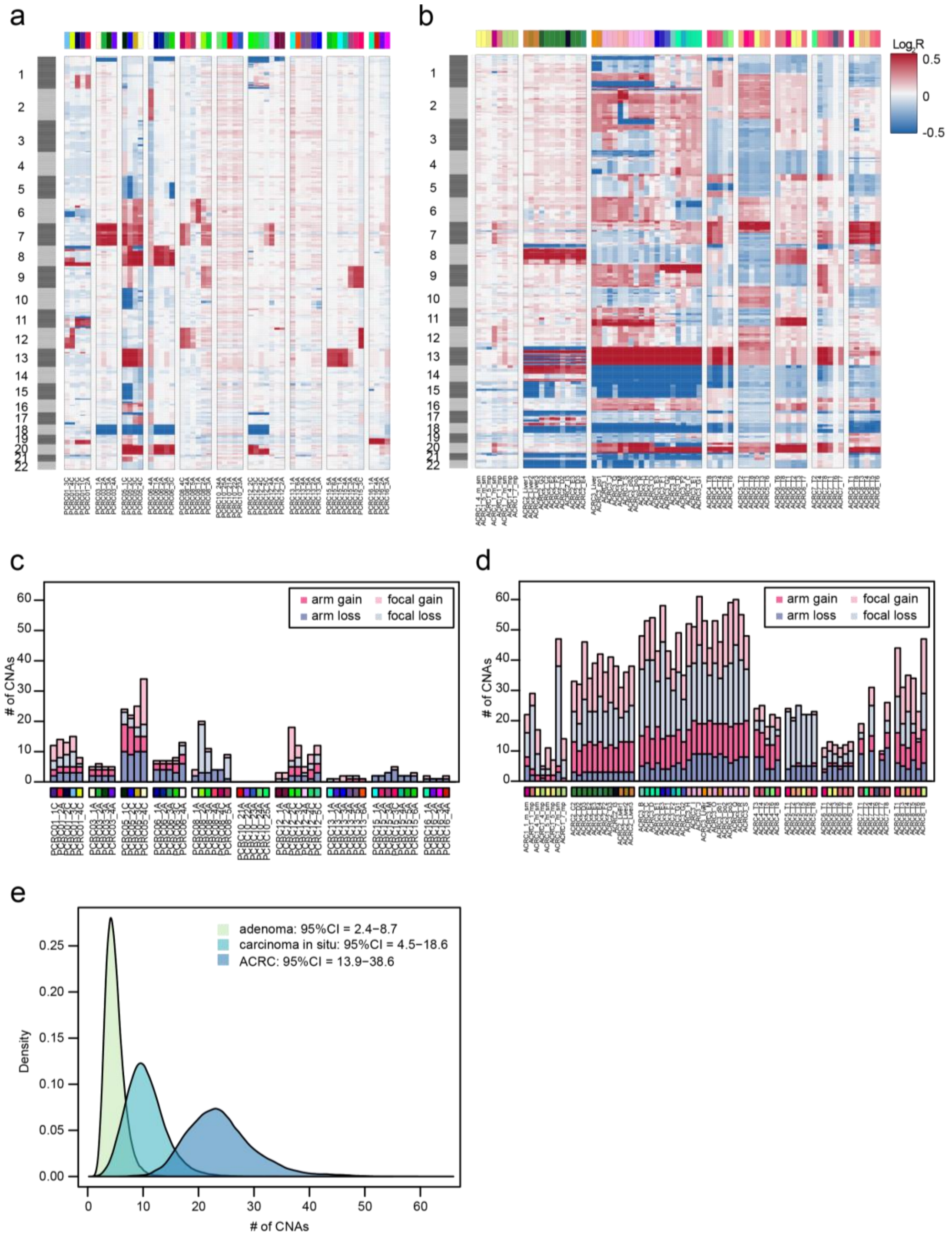

Supplementary Figure 11 CNA calling from our WES data. (**a**, **b**) Heat maps showing $\log_2$-scaled ratios between normalized tumor and normal read depths ($\log_2$R) calculated from our WES data of the 10 PCRCs (a) and eight ACRCs (b). Samples in each case are sorted in the same order as in **Fig. 1** and **Supplementary Fig. 2**. (**c**, **d**) Bar graphs showing the number of CNAs for each sample in the 10 PCRCs (c) and eight ACRCs (d). CNAs were categorized into four categories: arm-level gain (arm gain), arm-level loss (arm loss), focal gain and focal loss. (**e**) Comparison of the number of CNAs between three different stages: adenoma (n = 38) and carcinoma (n = 15) in PCRC, and ACRC (n = 70). Hierarchical Bayesian analysis was employed to remove the residuals associated with samples and cases (see Methods). The density plot represents an estimated posterior distribution of the corrected mean numbers of CNAs in adenoma, carcinoma in situ, and ACRC. Clear differences were observed between adenoma and carcinoma in situ, between carcinoma in situ and ACRC, and between adenoma and ACRC. 95% CI, 95 percent credible interval.

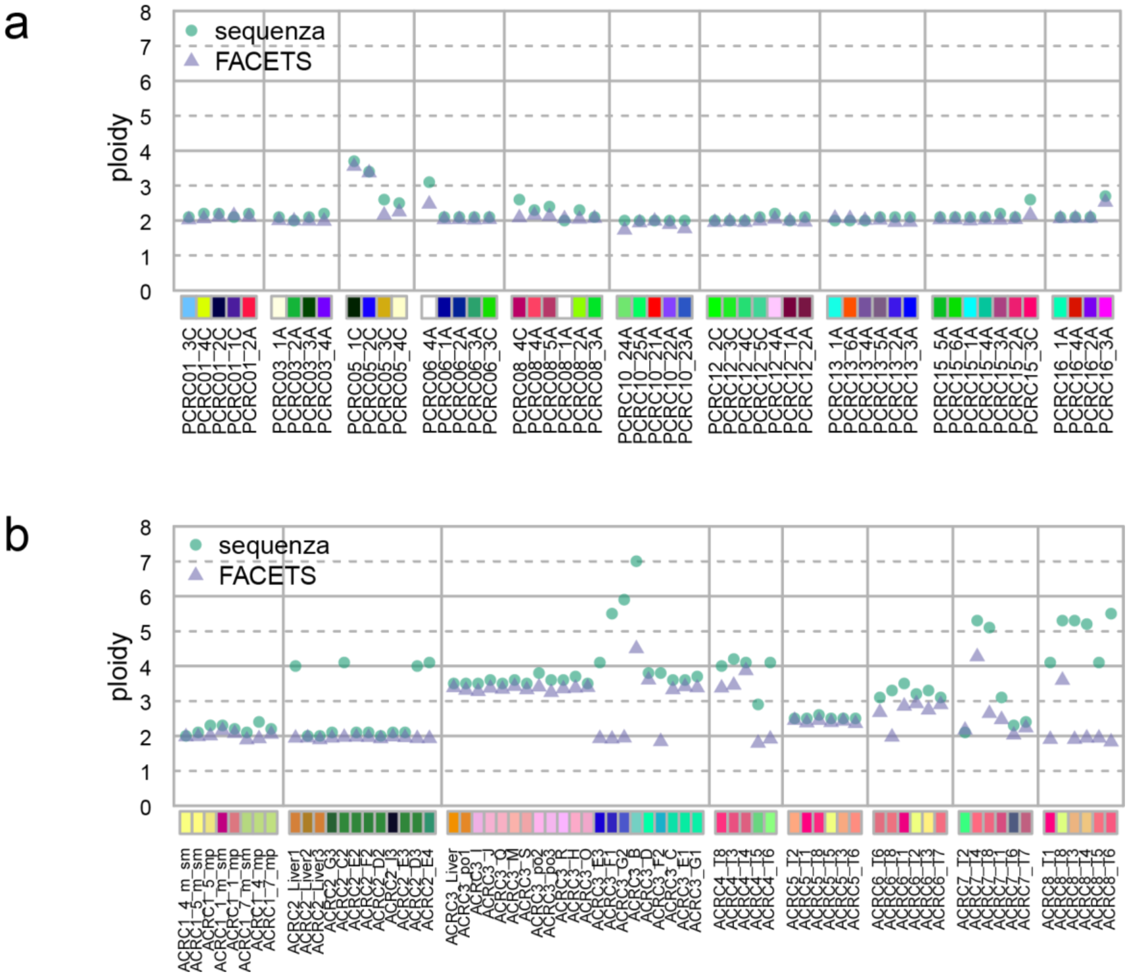

Supplementary Figure 12 Tumor ploidy profiling. (**a**, **b**) For each sample of the PCRC (a) and ACRC cases (b), tumor ploidy was estimated form WES data using two software tools, FACETS (light purple triangles; ref 47) and sequneza (light green circles; ref 48). The samples are sorted in the same order as **Fig. 1 and Supplementary Fig. 2.**

47. Shen, R. & Seshan, V.E. FACETS: allele-specific copy number and clonal heterogeneity analysis tool for high-throughput DNA sequencing. Nucleic Acids Res. 44, e131 (2016).

48. Favero, F. et al. Sequenza: allele-specific copy number and mutation profiles from tumor sequencing data. Ann. Oncol. 26, 64-70 (2015).

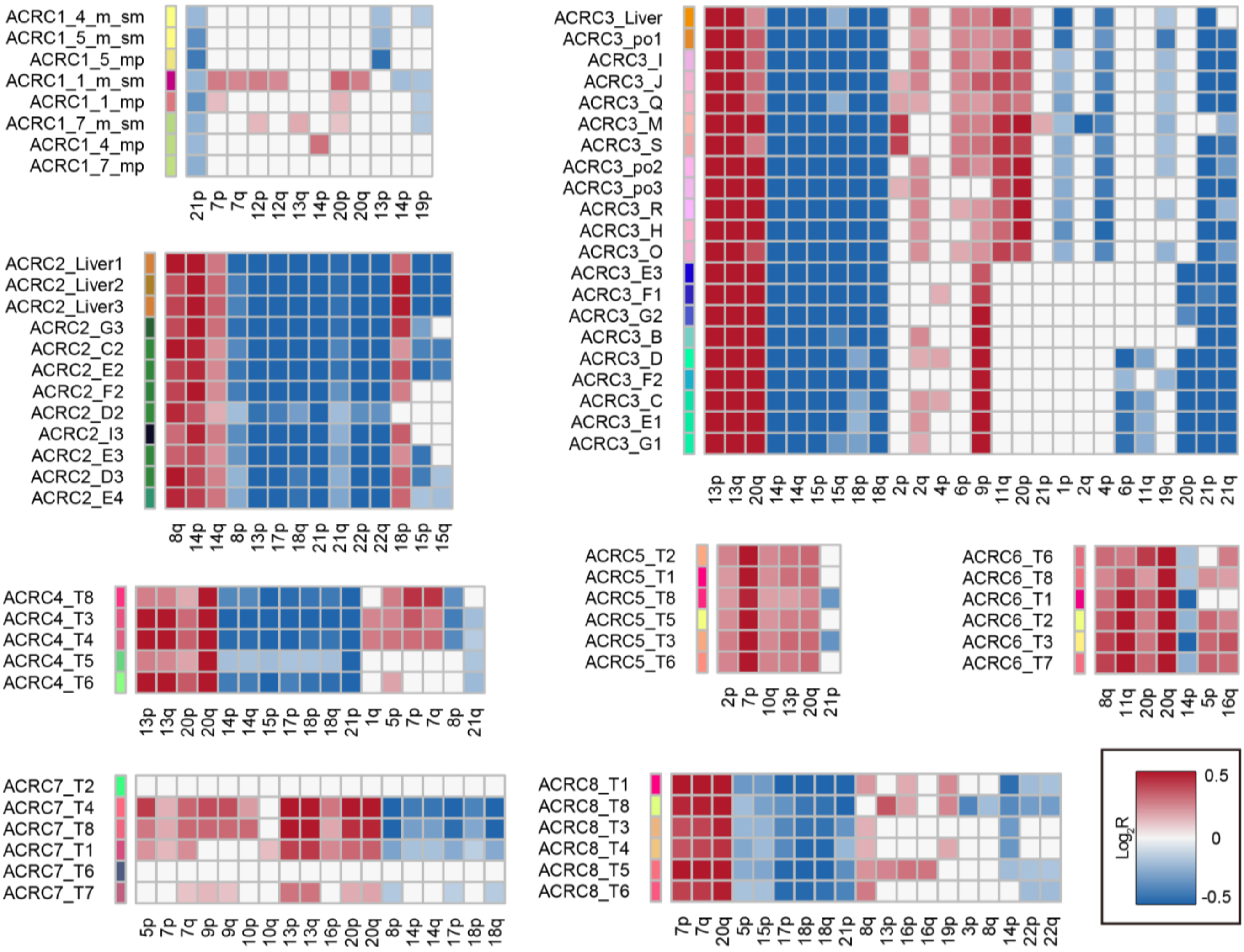

Supplementary Figure 13 Multiregion arm-level CNA profiles of ACRCs, presented as in **Fig. 4a**. Samples in each case are sorted in the same order as **Supplementary Fig. 2**.

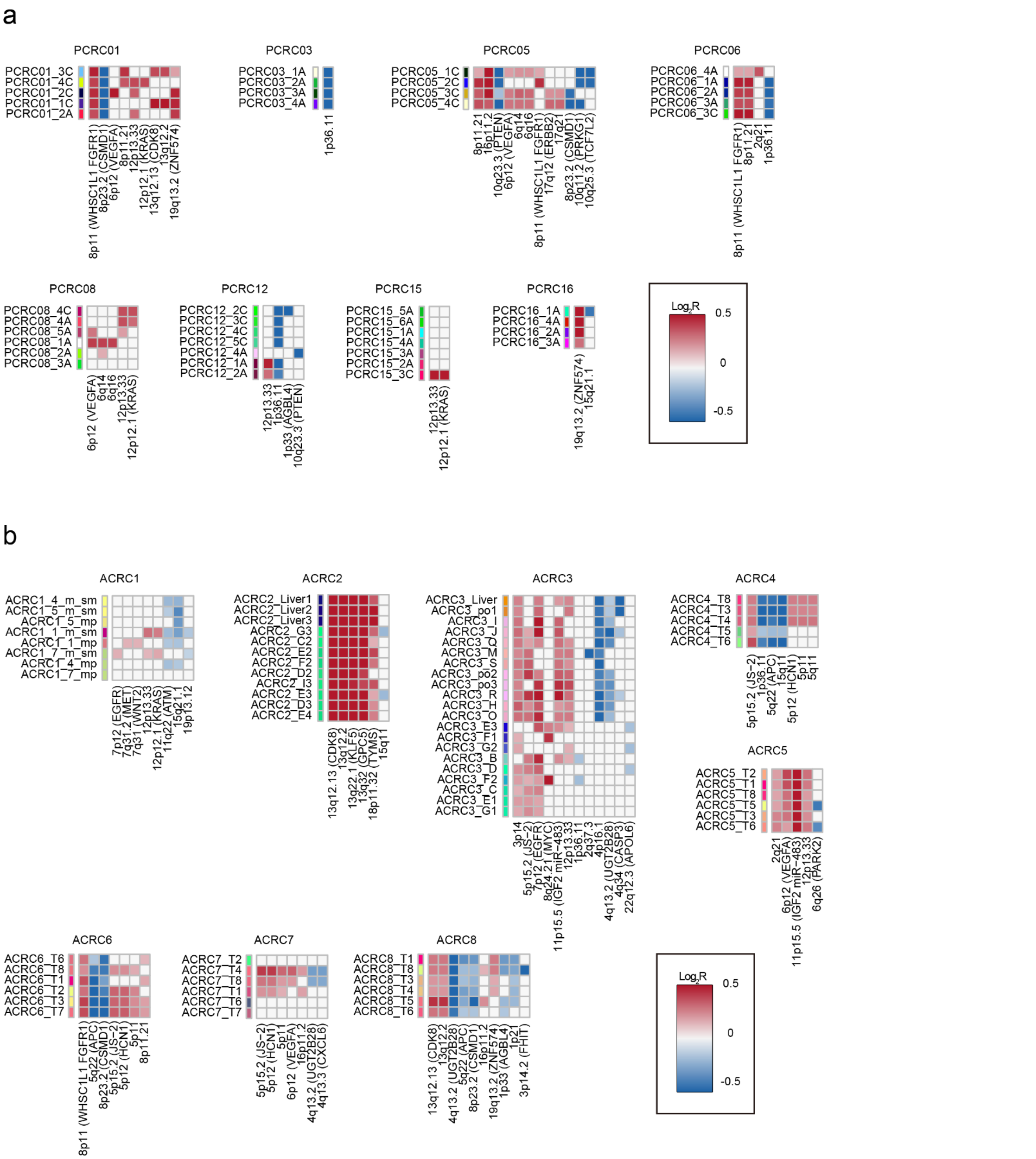

Supplementary Figure 14 Multiregion focal CNA profiles, presented as in **Fig. 4a**. (**a,b**) For each case of PCRC (a) and ACRC (b), we obtained multiregion focal CNA profiles by focusing on candidate loci that recurrently altered in previous reports (ref 19 and 26). Samples in each case are sorted in the same order as **Fig. 1 and Supplementary Fig. 2**. PCRC10 and PCRC13, in which no focal CNAs were detected, was omitted.

19. Cancer Genome Atlas, N. Comprehensive molecular characterization of human colon and rectal cancer. Nature 487, 330-337 (2012).

26. Wang, H., Liang, L., Fang, J.Y. & Xu, J. Somatic gene copy number alterations in colorectal cancer: new quest for cancer drivers and biomarkers. Oncogene 35, 2011-2019 (2016).

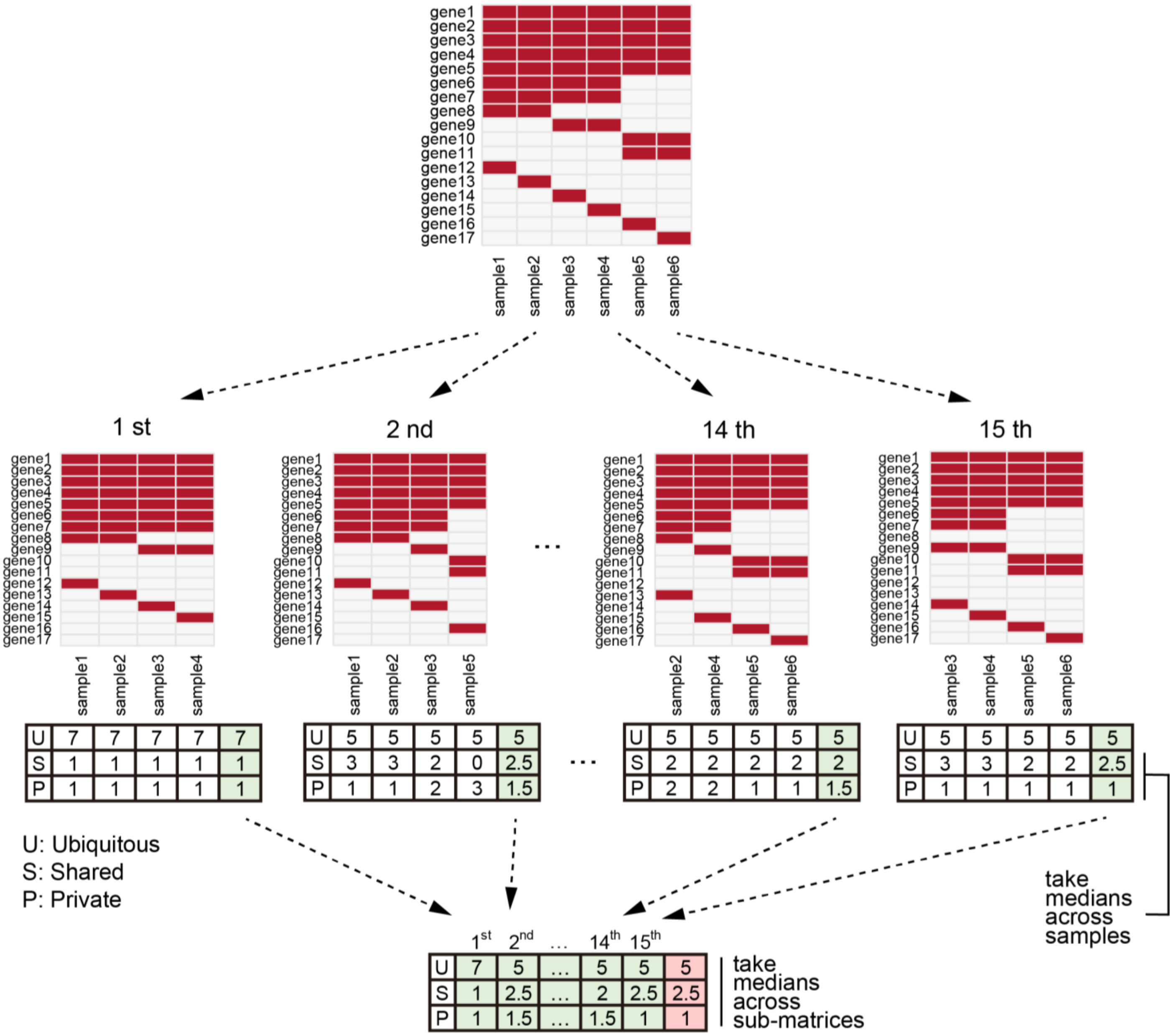

Supplementary Figure 15 An explanatory example of our down-sampling approach. This example shows a case of multiregion alteration profile matrix of six samples. (i) From the matrix, all 15 sub-matrices of four samples are obtained. (ii) For each of the sub-matrices, the numbers of alterations are counted for each of the 3 alteration categories: ubiquitous, shared and private. As for shared and private mutations, the median across samples are obtained since the numbers vary across samples in the same sub-same matrix. (iii) For each alteration category, the numbers of alterations are collected across the sub-matrices and their medians are finally obtained.

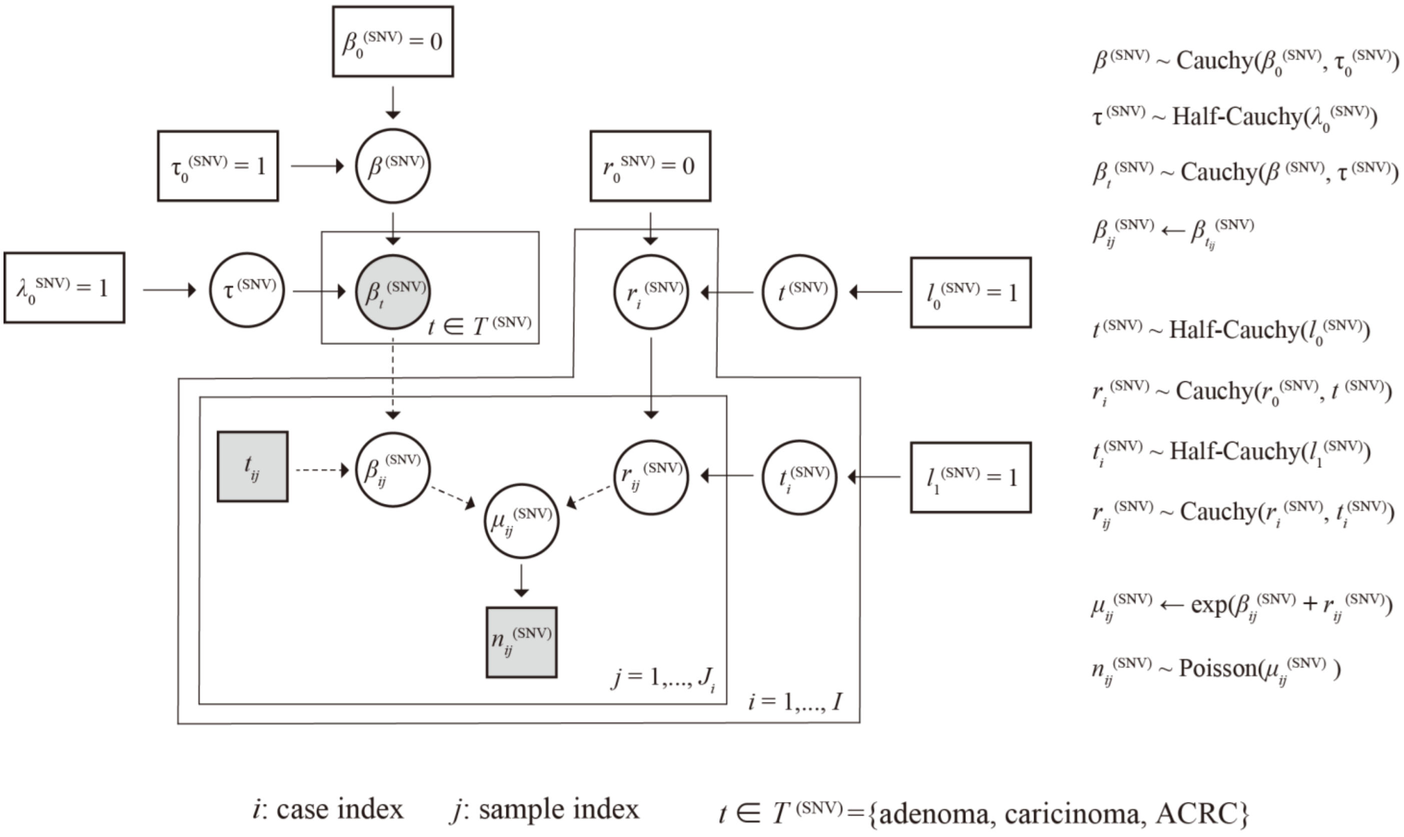

Supplementary Figure 16 Probabilistic graphical representation of the model used for comparison of the numbers of mutations between different tumor stages. Circles and rectangles represent random variables and fixed constants, respectively. Shaded squares indicate observed data while a shaded circle indicates a variable of interest. Solid and dashed arrows represent probabilistic and deterministic dependencies, respectively. For definition of variables, see Methods. SNV, single nucleotide variant.

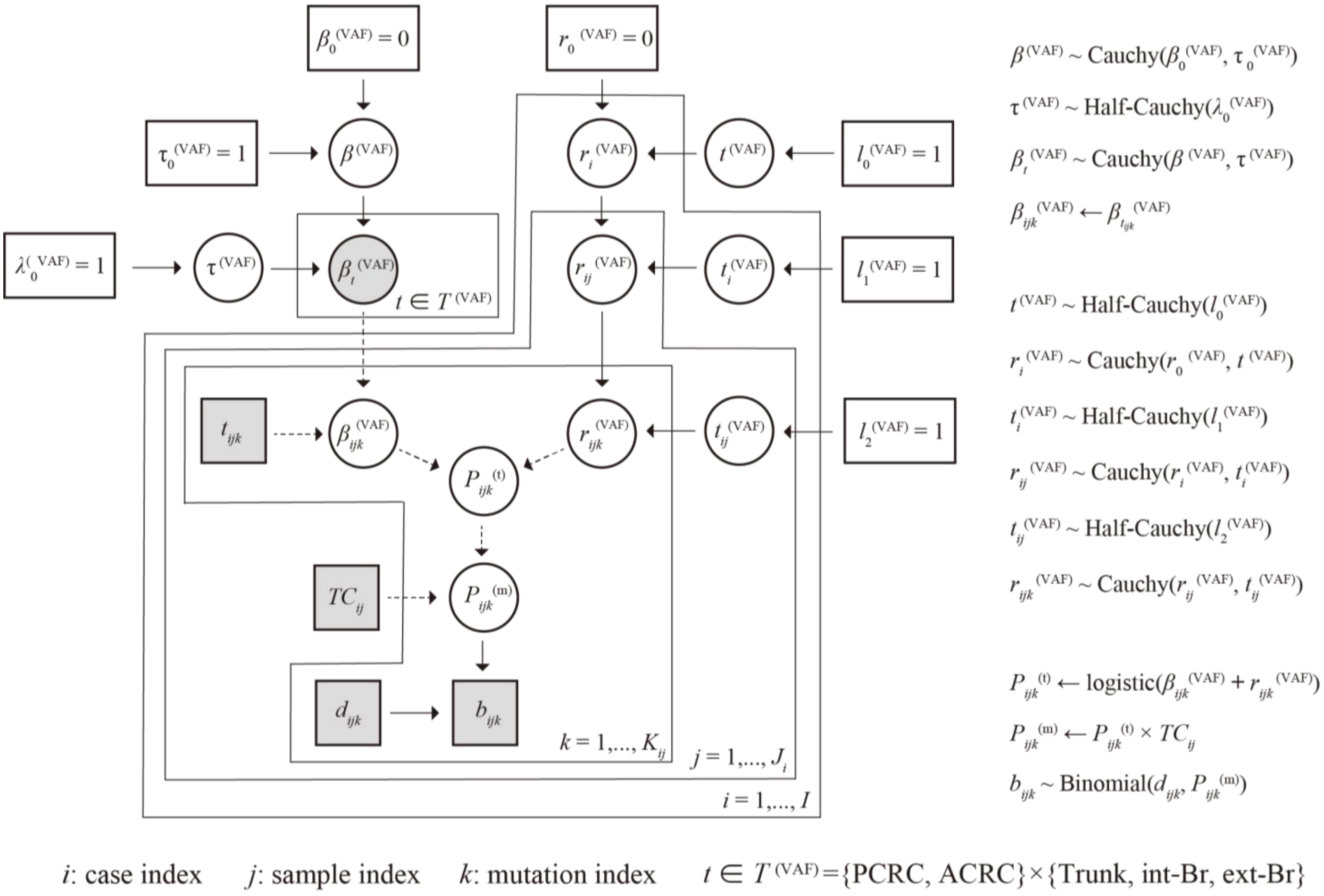

Supplementary Figure 17 Probabilistic graphical representation of the model used for comparison of variant allele frequencies (VAFs) between different categories of mutations, presented as in **Supplementary Fig. 16**. int-Br, internal branch; ext-Br, external branch.

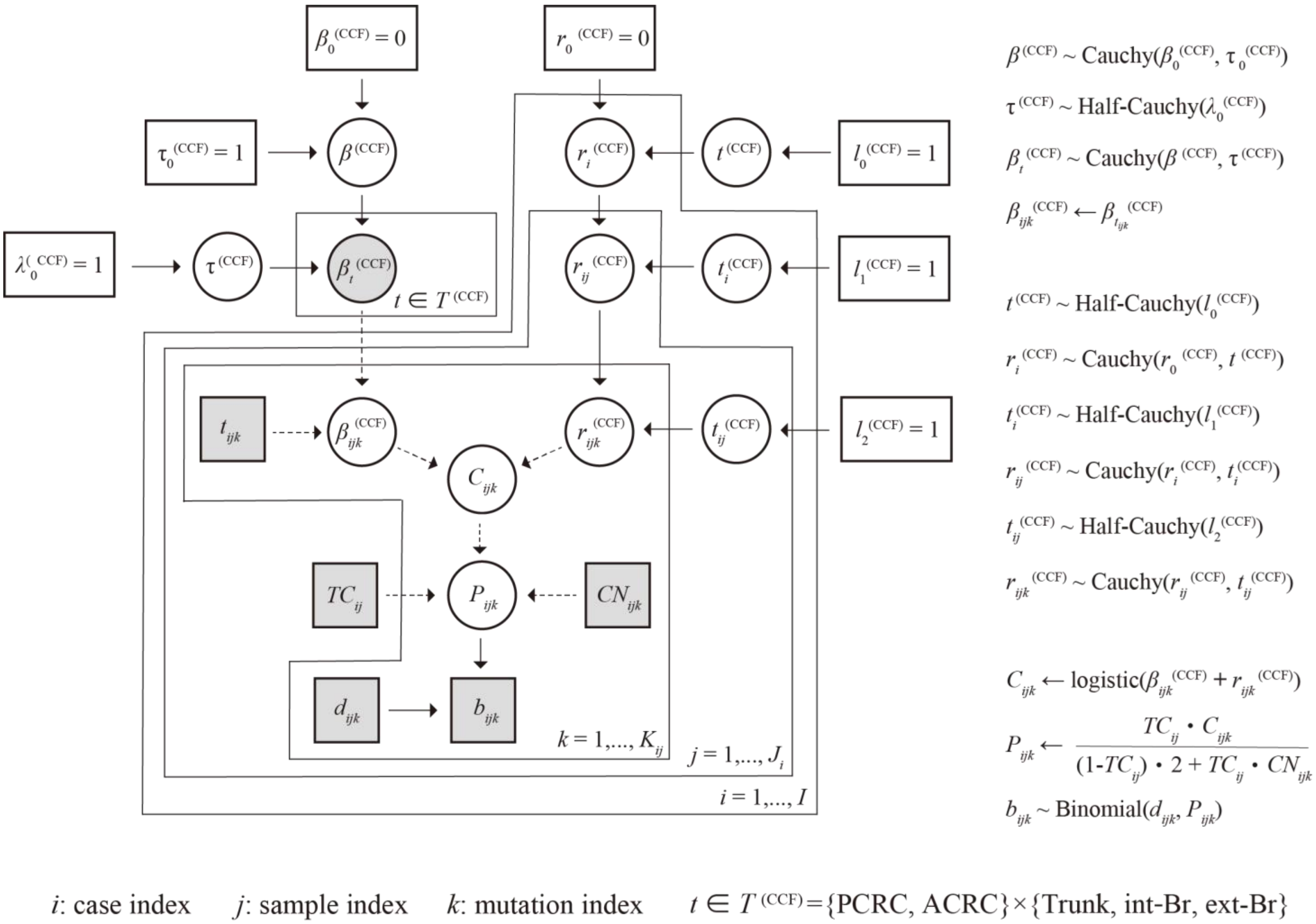

Supplementary Figure 18 Probabilistic graphical representation of the model used for comparison of cancer cell fractions (CCFs) between different categories of mutations, presented as in **Supplementary Fig. 18.** int-Br, internal branch; ext-Br, external branch.

Supplementary Tables

Supplementary Table 1. List of driver genes

gene	% of TCGA
APC	76.8
TP53	52.4
KRAS	43.8
PIK3CA	19.7
FBXW7	16.7
SMAD4	12.0
TCF7L2	9.9
NRAS	9.4
BRAF	9.4
ARID1A	8.6
SMAD2	6.9
ARID2	6.4
ACVR1B	6.0
ERBB3	6.0
AXIN2	6.0
CDC27	5.6
MAP2K4	4.7
CTNNB1	4.7
CASP8	4.3
ERBB2	4.3
BCLAF1	3.9
BCOR	3.9
ELF3	3.4
RBM10	3.4
IDH2	3.4
PTEN	3.4
NTN4	3.0
CNBD1	3.0
PCBP1	2.6
TRAF3	2.6
B2M	2.1
GOT1	2.1
CD70	2.1
TRIM23	1.7
SIRT4	1.7

Supplementary Table 2. Comparison of proportion of driver mutation between public data and PCRC

	TCGA (%)	TCGA (n=233)	PCRC (n=10)	fisher p-value
APC	76.8%	179	8	1.000
TP53	52.4%	122	1	0.009
KRAS	43.8%	102	7	0.117
PIK3CA	19.7%	46	3	0.425
FBXW7	16.7%	39	1	1.000
SMAD4	12.0%	28	1	1.000
TCF7L2	9.9%	23	3	0.079
NRAS	9.4%	22	2	0.257
BRAF	9.4%	22	1	1.000
ARID1A	8.6%	20	1	0.602
SMAD2	6.9%	16	2	0.165
ARID2	6.4%	15	1	0.499
ERBB3	6.0%	14	1	0.478
ACVR1B	6.0%	14	1	0.478
AXIN2	6.0%	14	0	1.000
CDC27	5.6%	13	0	1.000
