# Supplementary material for: A temporal shift of the evolutionary principle shaping intratumor heterogeneity in colorectal cancer
Source: Nat Commun. 2018 Jul 23;9:2884. doi: 10.1038/s41467-018-05226-0 (PMC6056524; doi:10.1038/s41467-018-05226-0)
Supplement: Supplementary file 2 — Description of Additional Supplementary Files [file 41467_2018_5226_MOESM2_ESM.pdf]

Description of Additional Supplementary Files

File Name: Supplementary Data 1
Description: Detailed information of cases and samples.

File Name: Supplementary Data 2
Description: Information of 63 analysed samples.

File Name: Supplementary Data 3
Description: Information of all somatic mutations.
